# The hyperbolic model for edge and texture detection in the primary visual cortex

**DOI:** 10.1186/s13408-020-0079-y

**Published:** 2020-01-30

**Authors:** Pascal Chossat

**Affiliations:** Université Côte d’Azur, Mathneuro, INRIA & CNRS, Valbonne, France

**Keywords:** Primary visual cortex, Neural field equations, Texture perception, Structure tensor, Pattern formation, Hyperbolic geometry

## Abstract

The modeling of neural fields in the visual cortex involves geometrical structures which describe in mathematical formalism the functional architecture of this cortical area. The case of contour detection and orientation tuning has been extensively studied and has become a paradigm for the mathematical analysis of image processing by the brain. Ten years ago an attempt was made to extend these models by replacing orientation (an angle) with a second-order tensor built from the gradient of the image intensity, and it was named the structure tensor. This assumption does not follow from biological observations (experimental evidence is still lacking) but from the idea that the effectiveness of texture processing with the structure tensor in computer vision may well be exploited by the brain itself. The drawback is that in this case the geometry is not Euclidean but hyperbolic instead, which complicates the analysis substantially. The purpose of this review is to present the methodology that was developed in a series of papers to investigate this quite unusual problem, specifically from the point of view of tuning and pattern formation. These methods, which rely on bifurcation theory with symmetry in the hyperbolic context, might be of interest for the modeling of other features such as color vision or other brain functions.

## Introduction

Since the discovery by David Hubel and Torsten Wiesel (Nobel prize 1981) of the tiling of the primary visual cortex V1 into columns of orientations [31], or hypercolumns, tremendous progress has been made in the physiological and functional exploration of this brain area, which is mainly dedicated to the integration of basic visual features of images in the visual field, such as contours, contrast, size (spatial frequency), ocular dominance, and color. A series of remarkable experiments have unveiled a significant part of the functional architecture of hypercolumns, and numerous works based on these results have been dedicated to giving a coherent description of the way in which the visual informations are treated in V1 [25]. An extensive survey can be found in [40]. This in turn has led to suggestion that this neuronal architecture implements a specific geometry, which was optimized through natural selection to efficiently process images at the basic level of V1. Although correlations seem to exist between hypercolumns of orientation, columns of ocular dominance, and color perception (blobs of cytochrome oxydase), a mathematical representation of these correlations is still unclear [2], and we concentrate on texture detection in the following.

The best understood feature is contour detection. Nearly 30 years after the discovery of the columnar structure of V1, evidence arose that columns themselves are organized around “pinwheels”, which are singular points at which all orientations are equally detected [5]. In hypercolumns pinwheels come by pairs of clockwise and anticlockwise variation of the orientation when turning around the pinwheel, which gives the lattice of pinwheels a cristalline structure reminiscent of spin glasses in physics [40]. It was also discovered that hypercolumns are interconnected through long range “lateral” fibers, with the property that neurons which detect a specific orientation in one hypercolumn are preferentially connected to neurons with the same orientation in other columns. Moreover this set of connections is not isotropic. Instead, the neural fibers tend to be aligned along a specific direction which is strongly correlated with the orientation itself [6]. These facts being put together allowed to identify V1, seen as a contour detector, with a fiber bundle whose base is the visual field and the fiber is the set of orientations “above” each small region in the visual field. In addition the lateral connections endow this bundle with a natural sub-Riemannian structure which allows V1 to compute “global” contours from local informations. This geometrical approach of contour detection in V1 has been exposed in [40, 44].

In parallel integro-differential neural field models of activity in cortical tissues (grey matter) have been proposed [48, 49]. Indeed in the context of large populations of neurons it would be prohibitive to apply the classical Hodgkin–Huxley equations or even a simplified version like the integrate and fire equations [26]. Instead, averaged membrane potentials over “small” populations are considered in the continuous limit, meaning that the fiber bundle of orientations is identified with $\mathbb {R}^{2}\times \mathbb {P}^{1}$ where $\mathbb {R}^{2}$ is the base plane (the “horizontal” extension of V1) and $\mathbb {P}^{1}$ is the projective line (a set of orientations) sitting “above” each point in $\mathbb {R}^{2}$. For convenience $\mathbb {P}^{1}$ is further identified with the circle $\mathbb {S}^{1}$ (mod*π*). Then the evolution of the neural field in this domain is described by a phenomenological integro-differential equation which we refer to as Wilson–Cowan equation, with the hope that it will capture the main qualitative behavior of the neuronal activity in V1. This equation was applied to the problem of orientation tuning, which is the enhancement within the columns of orientation of the orientation’s selectivity [3, 7]. The analysis here relies on bifurcation theory for neural fields defined on $\mathbb {S}^{1}$ (the “fiber” of orientation angles). By taking account of the interactions between columns of orientation, this extends to a problem of pattern formation in the full fiber bundle $\mathbb {R}^{2}\times \mathbb {S}^{1}$. This spatial extension was considered by Bressloff et al. [10] who developed a theory for geometric hallucinations reported by patients under various circumstances (like psychotropic drug ingestion). Spontaneous pattern formation as a Turing instability for neural fields in $\mathbb {R}^{2}$, hence not taking into account the orientation, had been first considered by Ermentrout [19] but the work in [10], which allows to build contours of the images, extended it considerably. More recently Citti and Sarti [42] considered the same model as in [10] but switching on an input in the equation to simulate the response of V1 to a stimulus.

Wilson–Cowan equation and the orientation tuning as a bifurcation problem will be recalled in Sect. 3 where basic principles of bifurcation theory with symmetry (or “equivariant bifurcation theory”) are also recalled. Although the algebraic tools in this theory are superfluous for the tuning of orientation problem, they simplify and help organizing the analysis in the spatially extended case [10] and become unavoidable when more “exotic” symmetries are considered, like those appearing in the models introduced in Sect. 4.

The challenge is to integrate more features in the model, in a way consistent with the orientation model and with the known experimental facts about the functional organization of V1. This comes back to replacing the fiber bundle $\mathbb {R}^{2}\times \mathbb {P}^{1}$ by $\mathbb {R}^{2} \times \varXi $ where *Ξ* is a “feature space” containing the set of orientations and endowed with a “nice enough” geometry to allow for analysis and computations.

Several theories have been proposed to extend the orientation model. In [43, 44] the scale of the receptive profile of each neuron is taken into account. Then $\varXi \sim \mathbb {S}^{1}\times \mathbb {R}$, where the first component is the circle of orientation angles (mod*π*) and the second component is the scale. Therefore the fiber bundle is 4-dimensional and the nice thing is that it inherits a natural symplectic structure (which extends the contact structure of the orientation model). This structure gives an elegant explanation of shape recognition by the brain [43].

Another model was proposed in [8] to take account of the spatial frequency. Indeed experiments have shown that neurons in V1 are also sensitive to the frequency of the gratings which are presented to the animal in the experimentations [18]. Some experimental studies suggest that in the hypercolumn the spatial frequency goes to a minimum at one pinwheel and to a maximum value at the other pinwheel. Under a suitable change of variables the authors expressed the feature space *Ξ* as a sphere where the polar angle is the orientation and the azimuthal angle is a normalized logarithmic function of the scale. Therefore the poles represent the pinwheels. This sphere is endowed with its natural Riemannian metric, so that $\varXi \sim \mathbb {S}^{2}$ with its isometry group $O(3)$. Now the orientation tuning problem is extended to account for frequency tuning and falls into the class of pattern formation on the sphere, which has been widely studied in other contexts (see [14, 28]).

The aim of this paper is to review an alternative way to extend the orientation model of hypercolumns, which was proposed ten years ago by [12]. The idea is to replace the orientation angle by a “Gaussian average” at a specified scale of the matrix $\nabla I \,{}^{t}(\nabla I)$ where ∇*I* is the gradient of the image intensity and ^*t*^ indicates the transpose matrix [4, 34]. This symmetric, positive definite matrix is called in computer vision the structure tensor or second-moment matrix. It accurately detects edges and corners, on the contrary to the gradient itself, which leads to ambiguous interpretations in certain cases. This will be explained in Sect. 4.1. The space $SPD(2) $ of structure tensors may therefore be a good candidate for the feature space *Ξ* when restricted to edge and texture detection.

The space $SPD(2)$ possesses a natural Riemannian structure with isometry group $GL(2,\mathbb {R})$, the group of real invertible matrices. As we shall see, this allows to further identify *Ξ* with $\mathbb {R}^{+}_{\ast }\times \mathbb {D}$, $\mathbb {D}$ being the Poincaré disc, or pseudo-sphere, equipped with its hyperbolic structure. The analysis of the Wilson–Cowan equation and its bifurcations in this hyperbolic geometry setting has been undertaken in a series of publications [12, 13, 20–22]. In this paper I give a synthetic presentation of these studies.

The concept of structure tensor is introduced in Sect. 4, as well as the geometry of the space of structure tensors and its reduction to the hyperbolic surface, of which the Poincaré disc is the most suitable representation for our purpose.

Section 5 is devoted to pattern formation, that is, bifurcation analysis from a homogeneous basic state, of the Wilson–Cowan equation in the space of structure tensors. Some elements about spectral and Fourier analysis in the Poincaré disc are first introduced. Then it is shown that bifurcation analysis can be performed in the same spirit as in the Euclidian case, but it is technically more involved. Three types of solutions are presented: bifurcation of periodic waves, bifurcation of periodic patterns (in a sense which will be explained), and bifurcation in a bounded subdomain of the Poincaré disc. Localized solutions (bumps) have also been studied in [23, 24], but the techniques are different and quite involved and will not be presented here.

The paper ends with a conclusion where the main results are summarized and commented.

## Hypercolumns of orientation and the functional architecture of V1

The signal (spikes of action potential) which is generated on the retina is preprocessed in an intermediate region of the brain called the Lateral Geniculate Nucleus (LGN). Then the outgoing neural fibers are projected to the occipital part of the brain, in a region called the primary visual cortex V1. This region is dedicated to a first treatment of the basic features of the image, such as orientations, contrast, spatial frequency, and color. Higher level visual zones *V*2, *V*3, etc., which surround V1, further refine this process and a complex, and not fully understood interaction between these successive zones (including the LGN) is constantly operating. Nevertheless, in order to understand the basic functioning of the visual cortex, it is relevant to simplify the landscape by assuming that V1 is disconnected from its higher level neighbors.

The following experimental facts have been well established. There have been many important contributions to the anatomical and functional exploration of V1. An extensive bibliography accompanies the description of these results in [40] (Chaps. 3 and 4), which has inspired the present section.

**Figure 1:**
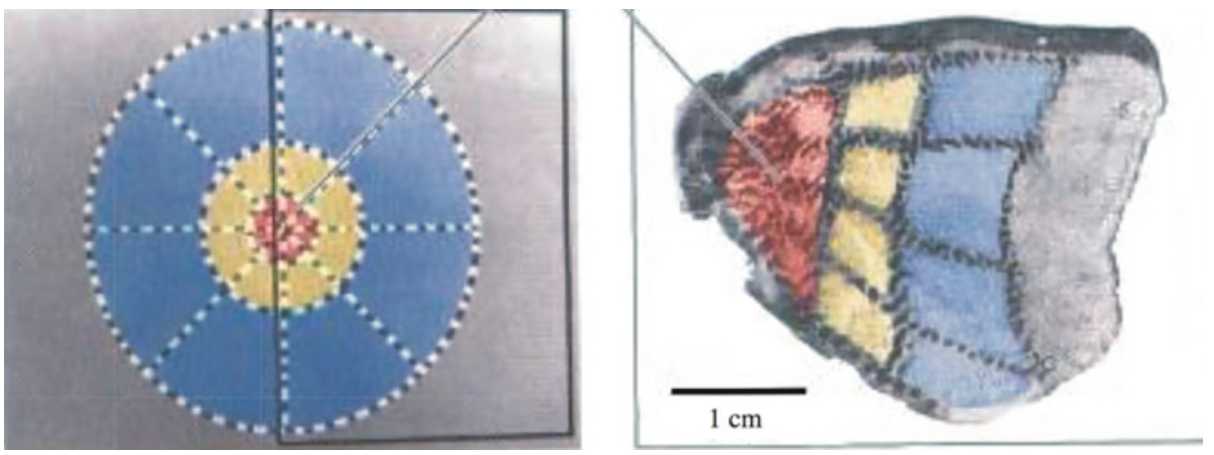
The retino-cortical map: left: the retina, right: the primary visual cortex. Colored regions on the left are projected to same color regions on the right (taken from [46], colored version)
- Each neuron in V1 responds to a specific small zone in the retinal plane called the *receptive field* of the neuron. This zone is generally elongated in a direction which defines the orientation to which the neuron is sensitive and the response of the neuron, called its *receptive profile* (RP), is well approximated by Gabor functions or derivatives of Gaussian functions [40]. Figure 2 shows an example of a RP.

- V1 is layered in six “horizontal sheets” of neural tissue. Most of the incoming fibers from the LGN arrive in layer 4. In the following we shall not differentiate these zones and we shall consider V1 as a 2D area.
- The fibers projecting the retina to V1 map the visual field in a conformal manner, called the *retinocortical map* (Fig. 1). A good approximation is given by the complex logarithm function $\mathbf {r}=\log z$.

**Figure 2:**
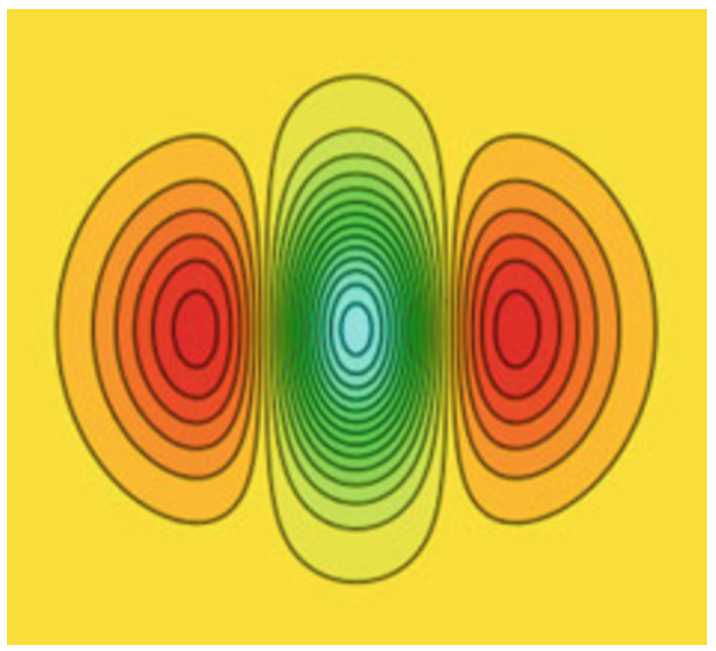
The typical receptive profile of a simple neuron in V1 (from [40]). Green color corresponds to a positive signal and red color to a negative signal
- The position and orientation to which neurons are responding in V1 are approximately constant in the “vertical” direction. This defines the *columns of orientation*. Since V1 is identified with a 2D area, each column of orientation corresponds to a point on this surface. Orientations vary continuously on the surface V1 except at singular points, called *pinwheels*, at which neurons respond to all orientations (their RP is circular). Along a small circle around a pinwheel all orientations that are detected within a small patch of the visual field are represented by steps of about 10^∘^. This property gives the distribution of pinwheels and orientations on V1 a crystalline structure as shown in Fig. 3. The elementary “cells” in this lattice is what we call *hypercolumns*, which incorporate pairs of pinwheels with reverse orientations. Iso-orientation lines converge to the pinwheels. Note that opposite rays correspond to orientations which differ by angle $\pi /2$.

**Figure 3:**
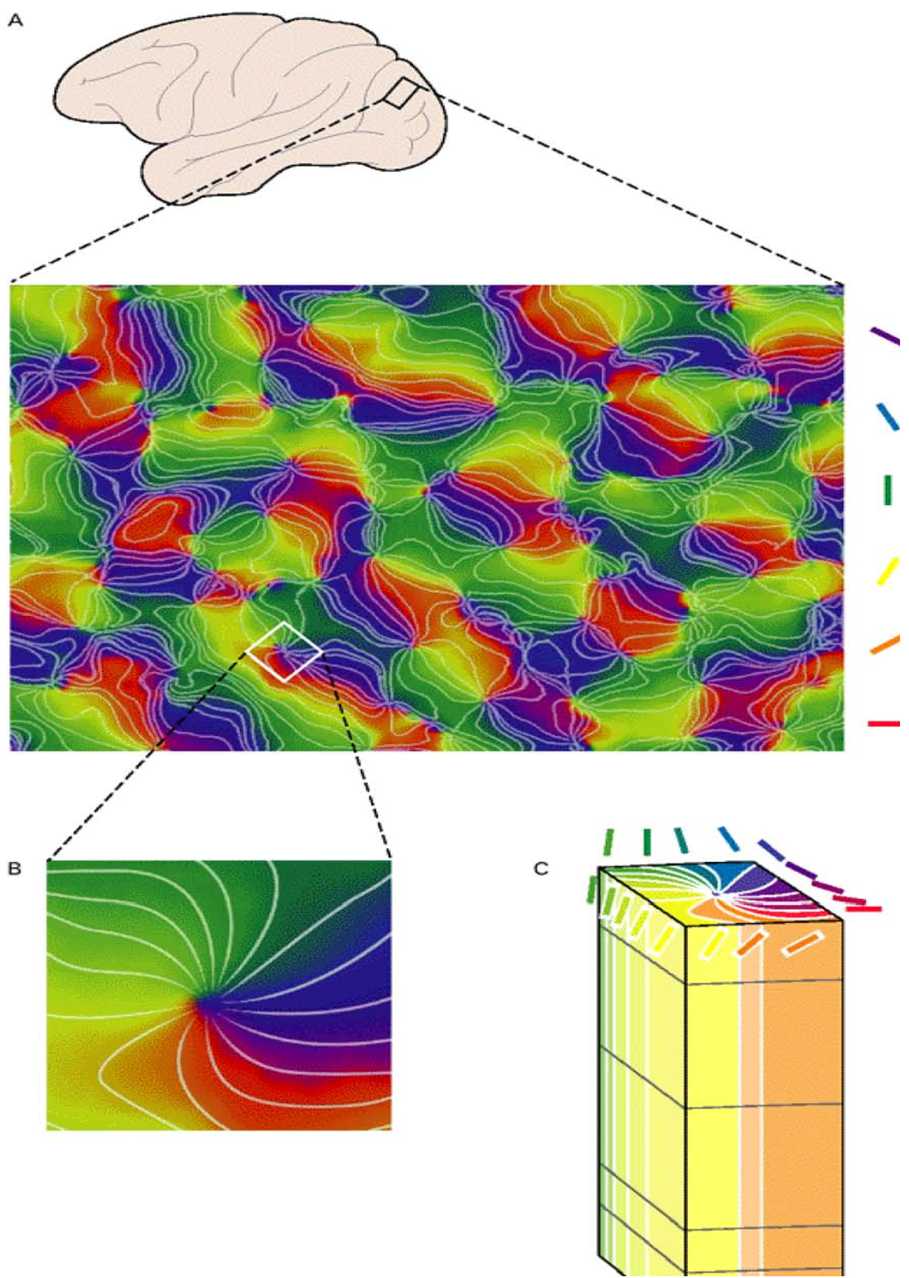
The crystalline structure of pinwheels and orientations in V1 (from [40]). Magnification shows a typical hypercolumn. Orientations are represented by colors. Iso-orientation lines (rays) are shown
- Long range connections between distant hypercolumns are selective in the following way. A neuron selective to one orientation *θ* in the hypercolumn projects fibers preferentially to neurons which are selective to the same orientation. Moreover, these connections tend to be aligned along a direction which, in a suitable frame centered on the pinwheel, is defined by the angle *θ*. This property is called *coaxiality*.

The lattice of pinwheels in V1 shows a remarkable regularity in first approximation [45]. If V1 is identified to a plane (in the “horizontal” directions), it can be well approximated by a periodic or a quasi-periodic lattice [7]. It is, however, a common procedure to replace this discrete structure by a continuous one, so that the “horizontal” extension of V1, which is homeomorphic to the visual field by retinotopy, is idealized by $\mathbb {R}^{2}$. Above each point in this plane the hypercolumn of orientation is modeled by the projective line $\mathbb {P}^{1}$ or, equivalently, by the circle $\mathbb {S}^{1}$ (mod*π*). The relevance of this idealization is discussed in [40]. Therefore V1 is now a fiber bundle $\mathbb {R}^{2}\times \mathbb {S}^{1}$ ($\mathbb {S}^{1}$ is more convenient than $\mathbb {P}^{1}$ for our purpose). It is a remarkable fact that the action of the group of rigid displacements $SE(2):=\mathbb {R}^{2}\ltimes SO(2)$ given by

1 $$ (\mathbf {r},\theta )\mapsto (R_{\varphi }\mathbf {r}+\mathbf {a},\theta + \varphi ), \quad (\mathbf {a}, \varphi )\in SE(2) $$

defines a *connection* (in geometrical sense) in $\mathbb {R}^{2}\times \mathbb {S}^{1}$, which is well adapted to the detection of contours of images and which, moreover, fits well with the neural connectivity in V1 that has been described above! There is a rich geometric structure associated with this, which has been exploited to analyze problems of visual perception such as contour continuation, illusory contours, visual hallucinations, detection of shapes [10, 15, 16, 42–44].

## Bifurcation of neural fields in V1: the tuning of orientations

### The neural field equations

In a celebrated paper [48], H.R. Wilson and J.D. Cowan stipulated more than 45 years ago that the cortex is fractioned into spatially localized zones into which neurons are densely connected to each other and dedicated to perform the same “task”. On the basis of a coarse-graining argument they replace each of these populations by a couple of differential equations, one for the mean firing rate $v_{E}(t)$ of excitatory neurons and the other for the mean firing rate $v_{I}(t)$ of inhibitory neurons. At a larger scale such local populations can be interconnected, so that the activity is represented by two scalar fields, one for the excitatory populations of neurons and one for the inhibitory populations. In the case of V1 the spatially extended domain is identified with the fiber bundle $\mathbb {R}^{2}\times {\varXi }$ where *Ξ* is a Riemannian space where each point represents a set of features of the image, for example, an angle *θ* (mod*π*) if one is exclusively interested in the orientation. The system can be further reduced to a single equation by assuming short-range excitation and longer range inhibition, which seems consistent with observations. Then the system of Wilson and Cowan reduces to a single integro-differential equation

2 $$ \frac{\partial v}{\partial t}(\mathbf {r},\xi ,t)=-\alpha v(\mathbf {r},\xi ,t) + \int _{\mathbb {R}^{2}} \int _{\varXi } w\bigl(\mathbf {r},\xi ;\mathbf {r}',\xi '\bigr) S_{\mu }\bigl(v\bigl(\mathbf {r}', \xi ',t\bigr)\bigr)\,d\xi '\,d\mathbf {r}' + I_{\mathrm{ext}}, $$

where the connectivity function $w(\mathbf {r},\xi ;\mathbf {r}',\xi ')$ models the synaptic strength (weight) from “neuron” $(\mathbf {r}',\xi ')$ to “neuron” $(\mathbf {r},\xi )$, so that $w(\mathbf {r},\xi ;\mathbf {r}',\xi ')$ takes a maximal and positive value when $(\mathbf {r},\xi )=(\mathbf {r}',\xi ')$, negative values when the two points are further apart, and is integrable at each point $(\mathbf {r},\xi )$. An important and natural assumption is that the form of *w* is consistent with the geometrical organization of the connectivity. This claim will be made more precise in Sect. 3.3.

The value $\alpha >0$ is the time constant of the units (localized population) whose activity decays in the absence of stimuli. It can be set to 1 by rescaling time, so that we will take $\alpha =1$ in all the following.

The function $S_{\mu }(x)$ is a sigmoid with limiting values 0 and 1. Roughly speaking it represents the fraction of neurons that are excited with activity *x*. The usual choice is $S_{\mu }(x)=(1+e^{-\mu (x- \kappa )})^{-1}$, where $\mu \geq 0$ is the “gain” of the transfer function $S_{\mu }$ and *κ* is a threshold value. Finally, the term $I_{\mathrm{ext}}$ is the resultant of external inputs to the system, which may be a function of **r** and *ξ* (but we always assume that it is time-independent).

Despite its highly simplified form, this equation has proven to be efficient in modeling the evolution of the coarse grained activity in V1, as we will see in Sect. 3.3. Before this, a short introduction to bifurcation theory is provided without proofs or technical details.

### A short overview of bifurcation theory

Let us rewrite Eq. (2) for neural fields in a domain $\mathcal {D}$ which is not specified for the moment but which is assumed to be a Riemannian space with volume element *d***x**:

3 $$ \frac{\partial v}{\partial t}(\mathbf {x},t)=-v(\mathbf {x},t) + \int _{\mathcal {D}} w\bigl(\mathbf {x};\mathbf {x}'\bigr) S_{\mu }\bigl(v\bigl(\mathbf {x}',t\bigr)\bigr)\,d\mathbf {x}' + I_{\mathrm{ext}}. $$

In this section we set $I_{\mathrm{ext}}=0$ (for simplicity). We assume that the connectivity kernel *w* is an integrable function in $\mathcal {D}$ and that it is invariant under a group of isometric transformations *G*: if $g\in G$, then $w(g\mathbf {x},g\mathbf {x}')=w(\mathbf {x},\mathbf {x}')$. For example, if $\mathcal {D}=\mathbb {R}^{2} \times \mathbb {S}^{1}$ as in Sect. 2, then $G\simeq SE(2)$ with the action defined by (1) (or $E(2)$ if reflections are included in the symmetries). Equation (3) is well defined and is Lipschitz continuous in the space $L^{\infty }(\mathcal {D})$ of bounded functions in $\mathcal {D}$, which is a Banach space with the sup norm. Then the Cauchy problem is well posed [22] (other functional settings with more regularity can be proposed, see e.g. [41] when $\mathcal {D}=\mathbb {R}^{n}$).

The invariance property of the kernel *w* implies that Eq. (3) is invariant under isometric transformations: for a function *f*, let $T_{g} f(\mathbf {x}) =f(g^{-1}\mathbf {x})$, then $g\mapsto T_{g}$ defines a linear representation of the group *G* in the space $L^{\infty }(\mathcal {D})$. Applying $T_{g}$ to (3) does not change this equation, which is said *G*-invariant.

Let us now assume that there exists a smooth family $v_{\mu }$ of *basic states* of (3): at each value of *μ*, $v_{\mu }$ is a stationary and homogeneous solution of (3). Homogeneous means that $v_{\mu }$ is invariant under the action of *G*: $T_{g} v_{\mu }=v_{\mu }$. In the cases we will consider here, this is equivalent to saying that $v_{\mu }$ does not depend on **x**. The basic state corresponds to the state of the rest of the neural network. It is sometimes set to $v_{\mu }=0$ by setting $S_{\mu }(0)=0$, possibly after a suitable translation [10]. It is asymptotically stable when the parameter *μ* is lower than a critical value $\mu _{0}$ [22]. Then the question is what happens when *μ* exceeds $\mu _{0}$. The couple $(\mu _{0},v_{\mu _{0}})$ is a *bifurcation point*.

The characterization of asymptotic stability of $v_{\mu }$ is that the linearized operator at $v_{\mu }$

4 $$ (L_{\mu }u) (\mathbf {x})=-u(\mathbf {x}) + \int _{\mathcal {D}} w\bigl(\mathbf {x};\mathbf {x}' \bigr)S'_{\mu }\bigl(v _{\mu }\bigl(\mathbf {x}'\bigr)\bigr)u\bigl(\mathbf {x}',t\bigr)\,d\mathbf {x}' $$

has its spectrum $\varSigma (L_{\mu })$ included in the half-plane $\{\lambda \in \mathbb {C}; \operatorname{Re}(\lambda )\leq \xi <0\}$.

In order to apply classical bifurcation theory, we now require that at $\mu =\mu _{0}$ the set $\varSigma _{c}=\varSigma (L_{\mu _{0}})\cap i\mathbb {R}$ consists of a finite number of isolated eigenvalues of finite multiplicity. When the isometry group *G* is non-compact, these conditions are in general not satisfied and the analysis should be restricted to some Banach subspace *V* of $L^{\infty}(\mathcal {D})$ in which *G* operates as a compact group [14]. $\varSigma _{c}$ is the center part of the spectrum. The corresponding solutions of the linearized equation at $v_{\mu _{0}}$

5 $$ \frac{\partial u}{\partial t}(\mathbf {x},t)=-u(\mathbf {x},t) + \int _{\mathcal {D}} w\bigl(\mathbf {x};\mathbf {x}'\bigr) S'_{\mu _{0}}\bigl(v_{\mu _{0}}\bigl(\mathbf {x}'\bigr)\bigr)u\bigl(\mathbf {x}'\bigr)\,d\mathbf {x}' $$

are called *critical* or *marginal modes*. They do not increase nor decay exponentially with time. Then it can be shown that (3) (with $I_{ext}=0$) satisfies the hypotheses of the center manifold theorem [14, 29]. The dynamics in a neighborhood of the bifurcation point is driven by the dynamics on a finite dimensional submanifold of *V*, the *center manifold*, the tangent space of which at $v_{\mu _{0}}$ is spanned by the critical modes. Let $V_{0}$ denote the space of these critical modes. The finite dimensional dynamics is then governed by an ODE in $V_{0}$, $\dot{x}=f(x,\mu )$, the asymptotic expansion of which can be computed by solving a sequence of Fredholm alternatives and changes of variables which put the system in its simplest form (called a *normal form*). Note that $f(0,\mu _{0})=0$ and the Jacobian matrix $f'_{x}(0,\mu _{0})$ has spectrum $\varSigma _{c}$. This ODE is the *bifurcation equation*. Its *μ*-dependent bounded solutions in a neighborhood of the bifurcation point define the bifurcated branches of solutions.

In general, with one single varying parameter one would expect that $\varSigma _{c}$ consists of simple eigenvalues and either $\varSigma _{c}=\{0 \}$ (steady-state bifurcation) or $\varSigma _{c}=\{\pm i\omega _{0}\}$ (Hopf bifurcation). However, symmetry can force higher multiplicity of the critical eigenvalues. Indeed the *G*-invariance of (3) implies that the operator $L _{\mu }$ is *equivariant*: for all $g\in G$, $L_{\mu }T_{g} =T_{g}L_{\mu }$ (the same holds for the nonlinear part of (3)). Therefore if *ζ* is an eigenvector (possibly a generalized eigenvector) for an eigenvalue in $\varSigma _{c}$, the same is true for $T_{g}\zeta $ for any $g\in G$: the space $V_{0}$ is *G*-invariant and its dimension is forced by the representation $T_{g}$ restricted to $V_{0}$.

In this short overview of bifurcation theory we focus on steady-state bifurcation. In this case the counterpart for 0 to be a simple eigenvalue of the *G*-equivariant linear operator $L_{\mu _{0}}$ is that the representation $T_{g}$ restricted to $V_{0}$ is *absolutely irreducible*: it is not decomposable into lower dimensional representations and, moreover, the only matrices that commute with $T_{g}$ in $V_{0}$ are real scalar multiples of the identity matrix. This property is *generic* for steady-state bifurcation problems with a scalar parameter [28]. It implies in particular that $V_{0}\simeq \ker (L_{\mu _{0}})$ (0 is a semi-simple eigenvalue). Another way to say it is that the critical modes do not depend on time.

The *G*-invariance property propagates to the center manifold and to the map $f(x,\mu )$, which is defined in $V_{0}$. This is a strong constraint on the structure of *f* and on its bifurcated solutions. One straightforward but tremendous consequence of this invariance is that branches of solutions can be classified by their *isotropy type*. Let *H* be an isotropy subgroup of *G*, that is, the largest group that fixes an element $x\in V_{0}$: $T_{h}x=x$, all $h\in H$. Then the set of elements in $V_{0}$ which are fixed by *H* is a linear subspace $V_{H}\subset V_{0}$. Moreover if $x_{0}\in V_{H}$, then the trajectory $x(t)$ with that initial condition for the bifurcation equation lies entirely in $V_{H}$. The isotropy type or orbit type of *H* is the conjugacy class of *H* in *G*, which is further identified with the set $G/H$. If a solution *x* has isotropy *H*, then its *G*-orbit, that is, the set of solutions $T_{g}x$, $g\in G$, is isomorphic to $G/H$.

As an example of application of these algebraic properties one has the following.

#### Theorem 1

(Equivariant branching lemma [28])

*For a generic one*-*parameter bifurcation problem withGsymmetry*, *there exists a branch of bifurcated solutions with isotropyHfor anyHsuch that* $\dim V_{H}=1$.

Indeed the equation $\dot{x}=f(x,\mu )$ restricted to $V_{H}$ is a scalar equation, and classical bifurcation theory at a simple eigenvalue applies to it. Moreover, $f'_{x}(0,\mu )=\sigma (\mu )\mathrm{Id}$ (Id is the identity matrix in $V_{0}$) by absolute irreducibility of the representation of *G*, hence the generic condition (for $\mu \in \mathbb {R}$) $\sigma '(\mu _{0})\neq 0$ is valid in any axis $V_{H}\subset V_{0}$.

#### Remark

The normalizer of *H* in *G*, denoted $N(H)$, is the largest subgroup which acts in $V_{H}$. In the case when $\dim V_{H}=1$, either $N(H)/H\simeq \{\mathrm{Id}\}$ and the branch of solutions can *transcritical* (two-sided) or $N(H)/H\simeq \{\pm \mathrm{Id}\}$ and the branch is always a *pitchfork*: the bifurcated solutions come by pairs of opposite signs in $V_{H}$ for either $\mu >\mu _{0}$ or $\mu <\mu _{0}$.

Much more can be said on this theory, especially when time-dependent solutions are considered. In any case the most important information to collect for systems which have symmetry is the way in which the symmetry group acts on critical modes. Then the equivariant branching lemma allows in many cases to determine the full bifurcation diagram. Note, however, that bifurcated branches, which have isotropy group not satisfying $\dim V_{H}=1$, may also exist; however they are computable by other means (like Morse lemma or degree theory, see [14] for a thorough exposition of this case).

### The tuning of orientations

The model and bifurcation methods introduced in the previous section have been applied to the tuning of orientation and spontaneous activation of patterns in V1 leading to visual geometric hallucinations [10, 19]. In this case $\mathcal {D}=\mathbb {R}^{2}\times \varXi $ with $\varXi \simeq \mathbb {S}^{1}$. In this section I survey these two problems, referring the interested reader to the bibliography for further details and discussion.

The tuning problem addresses the question of the sharpening in the hypercolumns of the signal arriving from the LGN with respect to orientation. There seems to be such a mechanism acting in the local cortical circuits (columns of orientation), and a simple model of this mechanism was proposed by [3] and [7]. Equation (2) should now be considered in its local version, that is, within a hypercolumn: the connectivity function *w* now depends only on the orientation angles (this is called the *ring model*) and (2) reduces to

6 $$ \frac{\partial v}{\partial t}(\theta ,t)=-v(\theta ,t) + \int _{\mathbb {S}^{1}} w\bigl(\theta ;\theta '\bigr)S \bigl(v\bigl(\theta ',t\bigr)\bigr)\,d\theta ' + I_{\mathrm{ext}}, $$

where the angular dependance of *v* and *w* is *π*-periodic. The question is: if the orientation input $I_{\mathrm{ext}}$ is broad, does the output *v* show a sharper distribution of orientations?

**Figure 4:**
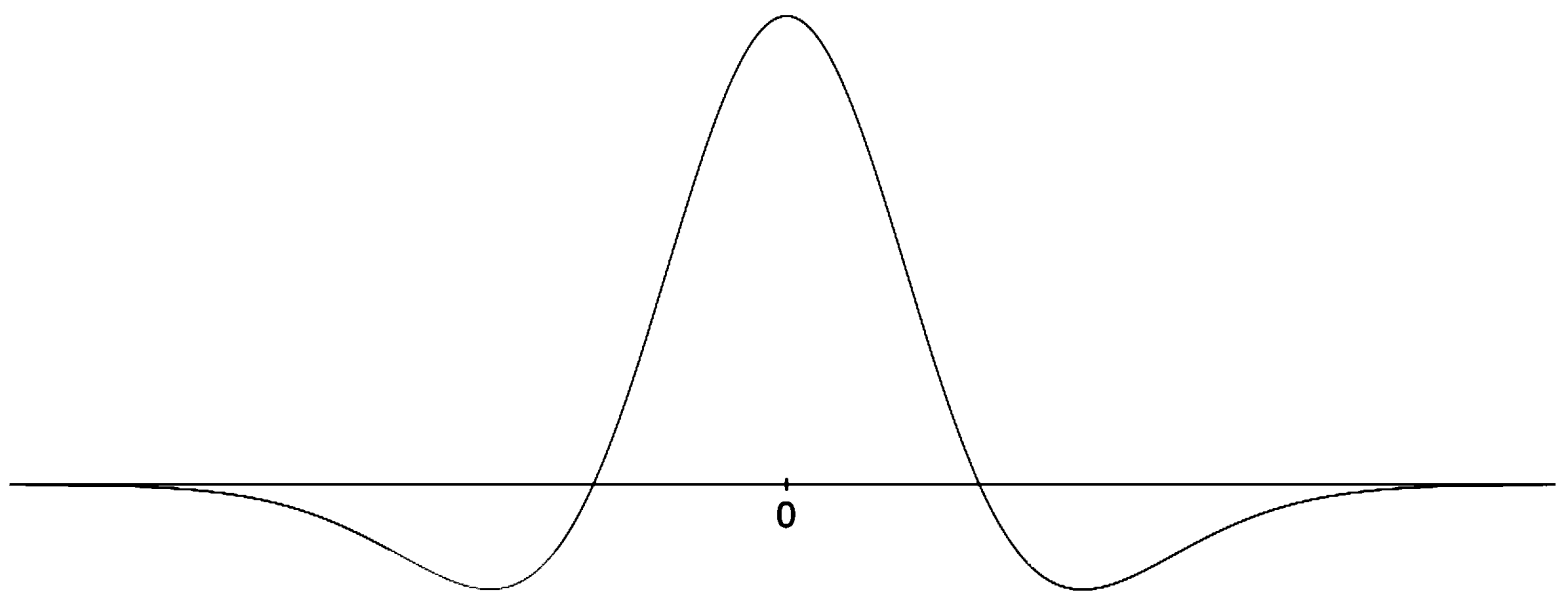
Example of a “Mexican hat” connectivity function

When the spatial extension is neglected, the isometry group $SE(2)$ reduces to $SO(2)$ acting in the fiber $\mathbb {S}^{1}$, with the constraint that angles 0 and *π* are identified: any function in (6) must satisfy $f(\theta + \pi )=f(\theta )$. Moreover, *w* should only depend on the distance $|\theta -\theta '|$ because there should not be an a priori preferred orientation (in fact, the connectivity within the hypercolumns shows a high degree of isotropy). It is usually assumed that the strength of the excitation is dominant when $\theta '$ is closer to *θ*, while the inhibition becomes dominant when $|\theta -\theta '|$ is larger. A model for this consists in taking $w(\theta ;\theta ')=h(\theta -\theta ')$ where *h* is the “Mexican hat” function

7 $$ h(x)=e^{-x^{2}/2\sigma _{1}}-Ae^{-x^{2}/2\sigma _{2}},\quad -\pi /2\leq x \leq \pi /2 $$

and constants are suitably chosen (typically $A<1$ and $\sigma _{1}< \sigma _{2}$, see Fig. 4). In order for a tuning effect to exist, it is necessary that in the limit when $I_{\mathrm{ext}}=0$ solutions to (6)–(7), which have broken *θ* invariance (they are not isotropic), exist and, moreover, are stable under small perturbations.

This is a typical bifurcation problem with symmetry group $G=O(2)$ because the fact that *w* only depends on the distance $|\theta - \theta '|$ implies that Eq. (6) is invariant with respect to the transformations $v(\theta )\mapsto v(\theta +\varphi )$ and $v(\theta )\mapsto v(-\theta )$.

The first step is to consider an isolated column of orientation: $I_{\mathrm{ext}}=0$. Then the form of *w* and $S_{\mu }$ implies that homogeneous steady states, solutions of the equation $v=\overline{w}S _{\mu }(v)$, where $\overline{w}=\int _{0}^{2\pi }h(s)\,ds$, always exist and are stable when *μ* is small enough. Such solutions, which we denote $v_{\mu }$, are $O(2)$-invariant, and since this group is compact, the method described in Sect. 3.3 applies.

The linearized operator at $v_{\mu }$ reads

8 $$ L_{\mu }u=-u +S'_{\mu }(v_{\mu }) \int _{0}^{2\pi }h\bigl(\theta -\theta '\bigr)u\bigl( \theta '\bigr)\,d\theta '. $$

Expanding $h(s)=\sum \hat{h}_{n}e^{2ins}$, $\hat{h}_{n}$ is real because *h* is an even function. Applying Fourier analysis. we obtain the real eigenvalues

9 $$ \lambda _{n}(\mu )=-1+S'_{\mu }(v_{\mu }) \hat{h}_{n} $$

with associated eigenfunctions $\cos (2n\theta )$ and $\sin (2n\theta )$. Therefore the eigenvalues are double except at $n=0$, for which they are simple (there is no symmetry-breaking in this case).

Note that $S'_{\mu }(v_{\mu })=\mu e^{-\mu (v_{\mu }-\kappa )}/(1+e ^{-\mu (v_{\mu }-\kappa )})^{2}$. Hence when *μ* is small enough all eigenvalues are negative and there exists a value $\mu =\mu _{c}$ at which the largest eigenvalue is $\lambda _{p}=0$ for a certain integer *p*, while all other eigenvalues remain negative.

Assuming $p>0$, this is an $O(2)$ symmetry-breaking steady-state bifurcation and $V_{0}$ is the space of critical modes $\{x\cos (2p \theta )+y\sin (2p\theta );(x,y)\in \mathbb {R}^{2}\}$. The bifurcation equation is an ODE in $(x,y)$. Instead of computing explicitly this equation and its bifurcated solutions, let us apply Theorem 1. Writing $z=x+iy$, the action of $O(2)$ in $V_{0}$ is spanned by rotations $R_{\varphi }:z\mapsto e ^{i\varphi }z$ and reflection $\kappa :z\mapsto \bar{z}$. Clearly $H=\{\mathrm{Id},\kappa \}$ is an isotropy subgroup such that $V_{H}=\mathbb {R}$ (the horizontal axis), hence the equivariant branching lemma applies. Moreover, rotations by $\pi /2$ transform the eigenvectors in their opposite, hence *x* in −*x*, which implies a pitchfork bifurcation. Remark that any axis in $\mathbb {R}^{2}$ is also an invariant axis and its orbit type is $G/H\simeq \mathbb {S}^{1}$. Therefore bifurcated solutions form circular orbits by the action of $SO(2)$ and, moreover, we have obtained all possible bifurcated solutions (if no additional degeneracy occurs in the problem).

The bifurcated branches can be either supercritical (i.e. for $\mu >\mu _{c}$) or subcritical. This depends on a coefficient which can be computed (at least numerically) from the bifurcation equation in $V_{H}$. The subcritical case gives rise to a hysteresis phenomenon: the branches bend back towards larger *μ* and the states of orientation tuning jump from the basic state $v_{\mu }$ to a well-developed solution “far” from bifurcation, see [7] for a discussion. From the orientation tuning point of view the most relevant case is when $p=1$, which corresponds to an unambiguously tuned response indicating the presence of a local contour.

When the external input is switched on with a specific orientation but a weak signal, the rotational symmetry is broken and it can be shown that the phase with that orientation is selected and the signal is sharpened [7], which explains the phenomenon of orientation tuning.

### Spontaneous activation of patterns in V1

The extension of the previous analysis to the spontaneous activation of the lattice of interconnected hypercolumns was undertaken by [10]. The following is a short exposition of the method which was used by [10]. Spontaneous activity means “in the absence of external input”, so we set $I_{\mathrm{ext}}=0$ in Eq. (2).

Now the variables are $\mathbf {r}\in \mathbb {R}^{2}$ and $\theta \in \mathbb {S}^{1}$ (mod*π*). Everything lies in the expression of the connectivity function $w(\mathbf {r},\theta ;\mathbf {r}',\theta ')$, which we decompose into the sum of a local term $w_{\mathrm{loc}}(\theta ,\theta ')$ (the ring model) and a term accounting for the long-range “lateral” connection $\beta w_{\mathrm{lat}}(\mathbf {r},\theta ;\mathbf {r}',\theta ')$. The coefficient *β* fixes the relative strength of the lateral connections. When $\beta =0$ (disconnected hypercolumns), *w* does not depend on spatial variables and the symmetry group of Eq. (2) is $E(2)\times O(2)$. It is biologically plausible to assume $\beta \ll 1$. It is also natural to assume that $w_{\mathrm{lat}}$ is invariant under the isometry group of the plane $\mathbb {R}^{2}$, but we have to account for the coaxiality property (1). Under these conditions the problem can be seen as a perturbation of the ring model, which has full symmetry $E(2)\times O(2)$, by a term which is invariant under coaxial transformations $v(\mathbf {r},\theta )\mapsto v(R_{\varphi }\mathbf {r}+\mathbf {a},\theta +\varphi )$. We also expect invariance by the reflection $v(\mathbf {r},\theta )\mapsto v(\bar{\mathbf {r}},-\theta )$, where $\bar{\mathbf {r}}=(r\cos \theta ,-r\sin \theta )$. Hence the symmetry group is now $G=E(2)$ acting in $\mathbb {R}^{2}\times \mathbb {S}^{1}$ via these transformations. A model of function $w_{\mathrm{lat}}$ satisfying these constraints was introduced in [10].

The bifurcation analysis proceeds along the same lines as exposed in Sect. 3.2 with the important difference that now the group *G* is not compact. If $\beta =0$, then the spectral analysis is similar to that of the ring model with the additional property that since in this case the equation does not depend on **r** any distribution of orientations amongst $\mathbb {R}^{2}$ (i.e. on V1) is possible. When $\beta >0$ the spectral analysis of the linear operator $L_{\mu }$, which now incorporates the kernel $w_{\mathrm{lat}}$, proceeds in looking for linear modes of the form

10 $$ \zeta (\mathbf {r},\theta )=u(\theta -\varphi )e^{i\mathbf {k}\cdot \mathbf {r}}+ \mathrm{c.c.} $$

with $\mathbf {k}=k(\cos \varphi ,\sin \varphi )$. Because of translational invariance, any wave vector **k** is allowed. Moreover, rotational invariance implies that the functions *u* do only depend on the wave number $\|\mathbf {k}\|=k$ and not on the direction *φ*. Therefore not only the spectrum is continuous but the eigenvalues have infinite multiplicity. The computation of the critical modes and the determination of the bifurcation point are not straightforward. They were undertaken in [10] by perturbation analysis with respect to *β* and I leave the interested reader to consult this reference for the details.

In the perspective of this review, what is important is the way to reduce the problem to one that can be handled with the bifurcation method introduced in Sect. 3.2. The idea, which goes back to Turing for pattern formation [47], is to look for solutions which are doubly periodic in $\mathbb {R}^{2}$, that is, which satisfy the condition $f(\mathbf {r}+\mathbf {a}+\mathbf {b})=f(\mathbf {r})$ with **a** and **b** not colinear. Let $\mathcal {L}=\{m\mathbf {a}+n\mathbf {b};m,n\in \mathbb {Z}\}$ be the lattice subgroup of $\mathbb {R}^{2}$ spanned by **a** and **b**. For (10) this comes back to retaining only those wave vectors **k** such that $\mathbf {k}\cdot \mathbf {a}=2m\pi $ and $\mathbf {k}\cdot \mathbf {b}=2n \pi $ with $m,n\in \mathbb {Z}$. Now doubly periodic functions can be seen as functions which are defined on the torus $\mathbb {R}^{2}/\mathcal {L}$, so that now the domain of the Wilson–Cowan equation becomes $\mathcal {D}=\mathbb {R}^{2}/\mathcal {L}\times \mathbb {S}^{1}$. The symmetry group acting on this space is $G=\mathbb {R}^{2}/\mathcal {L}\ltimes \mathrm{D}_{p}$, where $\mathrm{D}_{p}$ is the dihedral group of order 2*p* (called the holohedry of the lattice, see [36]). Here there are only three possibilities, depending on the angle between **a** and **b**: $p=2$ (rhombic lattice), $p=4$ (square lattice), and $p=6$ (hexagonal lattice). These groups are compact and the spectral analysis leads to isolated eigenvalues with finite multiplicities. In each case several absolutely irreducible representations of *G* exist, which leads to different critical spaces $V_{0}$ and different sets of isotropy types. The overall picture is rather complicated, but the remarkable result is that the bifurcated patterns, once transformed to retina images through the inverse of the retinotopic map, show a striking similarity with geometric hallucinations drawn by patients under various types of nonvisual stimulations (psychotropic drugs…). The group theoretical point of view of this problem has led to further developments, see [9, 27].

## The structure tensor model

### A texture detector and its implementation for V1

Despite its success, the ring model and its spatial extension described in the previous sections are incomplete in the sense that they account for only one (although essential) feature of images, namely the orientation of contours. In order to enhance the image texture processing, specialists of computer vision use a mathematical object named *structure tensor*, which is a $2\times 2$ symmetric, positive definite matrix. Its interest lies in the fact that not only the eigendirection associated with the smallest eigenvalue gives the orientation of the image, but also its two positive eigenvalues characterize the degree of inhomogeneity and contrast of the image at a given scale. The structure tensor is defined as follows. In all the following we denote $\mathbf {x}=(x,y)$ in canonical coordinates in $\mathbb {R}^{2}$ and

$$ g_{\sigma }(\mathbf {x})=\frac{1}{2\pi \sigma ^{2}}e^{-\frac{x^{2}+y^{2}}{2 \sigma ^{2}}} $$

is the 2D Gaussian function with standard deviation *σ*.

Let *I* be the image intensity and $I_{\sigma _{1}}=g_{\sigma _{1}} \ast I$ (convolution product) be the intensity function filtered (smoothed) at the scale $\sigma _{1}$. Then the structure tensor at **x** is the matrix

11 $$ \mathcal {T}(\mathbf {x})= \begin{bmatrix} g_{\sigma _{2}}\ast (I_{x}^{\sigma _{1}})^{2} & g_{\sigma _{2}}\ast I _{x}^{\sigma _{1}} I_{y}^{\sigma _{1}} \\ g_{\sigma _{2}}\ast I_{x}^{\sigma _{1}} I_{y}^{\sigma _{1}} & g_{\sigma _{2}}\ast (I_{y}^{\sigma _{1}})^{2} \end{bmatrix}, $$

where subscripts *x* and *y* denote the partial derivatives w.r.t. these variables. The parameter $\sigma _{2}$ relates to the size of the receptive field of neurons and to the characteristic scale at which the texture is seen [12]. Note that $\mathcal {T}$ has the form

$$ \mathcal {T}= \begin{pmatrix} a & c \\ c & b \end{pmatrix}, \quad a>0,ab-c^{2}>0. $$

Indeed $ab-c^{2}\geq 0$ by the Cauchy–Schwarz inequality and, moreover, the eigenvalues are strictly positive due to the spatial averaging which distributes the information of the image over a neighborhood. Therefore the set of structure tensors is identical to $SPD(2)$, the set of symmetric positive definite matrices, which is an open cone in $\mathbb {R}^{3}$.

Let $\lambda _{1}\geq \lambda _{2} > 0$ be the eigenvalues of $\mathcal {T}(\mathbf {x})$ and $\mathbf {e}_{1}\perp \mathbf {e}_{2}$ be the corresponding eigenvectors. Elementary algebra shows that

12 $$ \mathcal {T}=(\lambda _{1}-\lambda _{2}) \,{}^{t}\mathbf {e}_{1}\mathbf {e}_{1}+\lambda _{2}{\mathbf{I}} _{2}, $$

where ${}^{t}\mathbf {e}_{1}\mathbf {e}_{1}$ is the tensor product of $\mathbf {e}_{1}$ by its transpose. This has the following informative consequences for the image at scale $\sigma _{2}$:

- The size of $\lambda _{j}$ defines the variation of the intensity, hence the contrast, along the direction $\mathbf {e}_{j}$;
- if $\lambda _{1}\approx \lambda _{2}$, then the image is isotropic;
- if $\lambda _{1}\gg \lambda _{2}\approx 0$, then an edge is detected along the direction $\mathbf {e}_{2}$;
- if $\lambda _{1}\geq \lambda _{2}\gg 0$, then a corner is likely to be present;
- $\lambda _{1}-\lambda _{2}$ measures the degree of anisotropy of the image. The coefficient $\kappa =\frac{\lambda _{1}-\lambda _{2}}{\lambda _{1}+\lambda _{2}}$ is called the *coherence* of the image.

**Figure 5:**
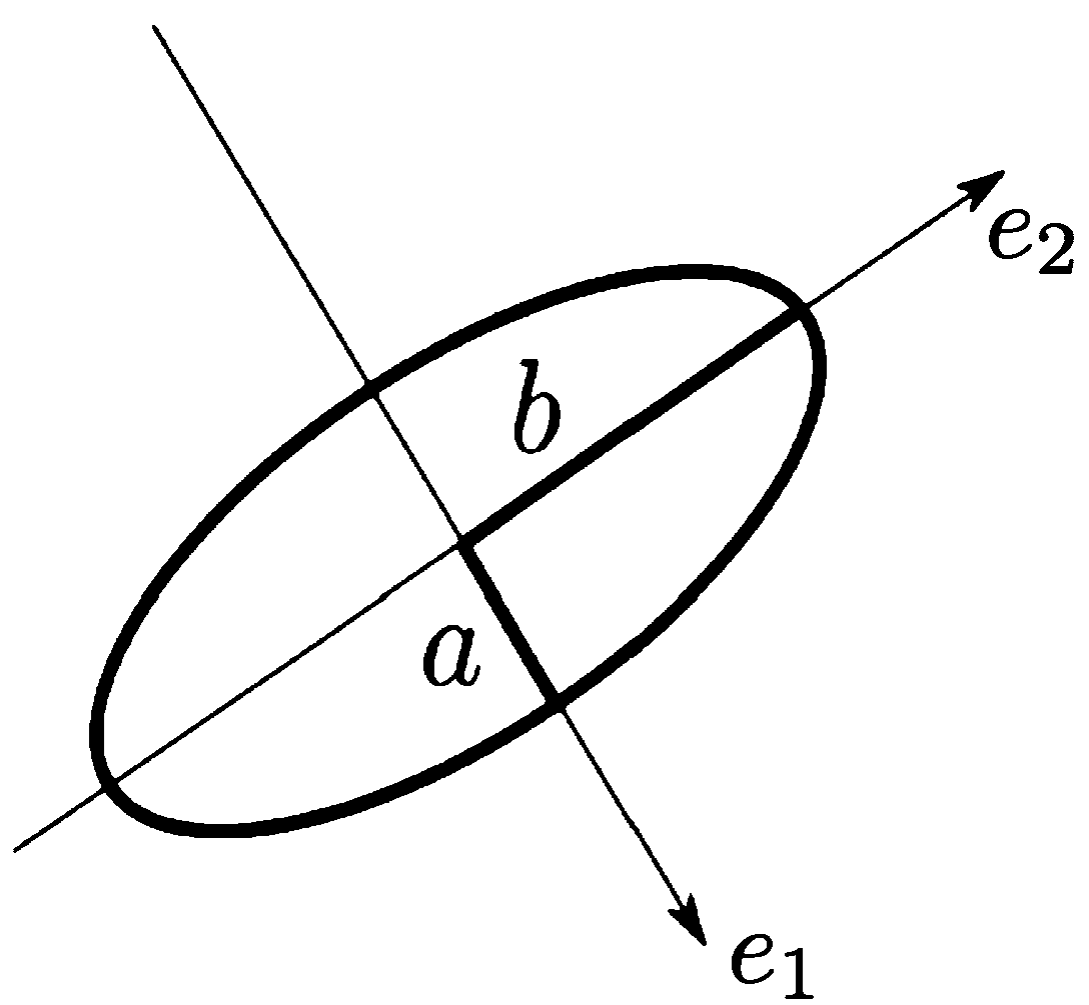
The ellipse $\mathbf {x}\mathcal {T}\,{}^{t}\mathbf {x}=1$. Semi-axes $a=1/\sqrt{\lambda _{1}}$, $b=1/\sqrt{\lambda _{2}}$

These properties are synthetized by drawing the locus of points ${}^{t}\mathbf {x}\mathcal {T}\mathbf {x}=1$ (Fig. 5). In [12] it was hypothesized that the structure tensors at one point **x** of the surface of V1 is encoded in the corresponding hypercolumn. Although there has been no experimental testing of this hypothesis yet, this idea is supported by the strong evidence that quantities related to image derivatives are represented in the neural activity [11, 40].

The aim now is to take $\varXi =SPD(2)$ in Eq. [48] as a generalization of the orientation model. The first idea would be to consider $SPD(2)$ as the cone embedded in $\mathbb {R}^{3}$ with its Euclidean metric. It would then be difficult to conciliate this choice with the natural assumption that neurons do not know in which coordinate system the components of the structure tensor are expressed. In other words Eq. (2) should be invariant under coordinate changes in the space of positive definite quadratic forms, in particular the connectivity function should share this property. In mathematical terms $SPD(2)$ is isomorphic to the homogeneous space $GL(2)/O(2)$ where $GL(2)$ is the group of invertible real matrices, which acts on $SPD(2)$ by $G\cdot \mathcal {T}= \,{}^{t}G\mathcal {T}G$. This induces a metric on the tangent space, defined by $g_{\mathcal {T}}(A,B)=\operatorname{tr}(\mathcal {T}^{-1}A\mathcal {T}^{-1}B)$, and the corresponding distance is

13 $$ d_{0}\bigl(\mathcal {T},\mathcal {T}'\bigr)=\sqrt{\log ^{2}\sigma _{1}+\log ^{2}\sigma _{2}}, $$

where $\sigma _{1}$, $\sigma _{2}$ are the eigenvalues of $\mathcal {T}^{-1}\mathcal {T}'$, see [37, 39] for details. Hence $GL(2)$ is the group of isometries of $SPD(2)$.

If we now restrict the analysis to the activity inside one hypercolumn, hence discarding lateral interactions like in the ring model, then $\mathbb {S}^{1}$ must be replaced by $GL(2)$ in (6) and the connectivity function must satisfy the invariance relation $w(\mathcal {T};\mathcal {T}')=w(g\cdot \mathcal {T},g\cdot \mathcal {T}')$ for all $g\in GL(2)$. Extending the domain to V1 requires to take account of the anisotropy of lateral connections. Like in the planar case (see Sect. 3.3) $w(\mathbf {x},\mathcal {T};\mathbf {x}',\mathcal {T}')=w_{\mathrm{loc}}(\mathcal {T},\mathcal {T}')+ \beta w_{\mathrm{lat}}(\mathbf {x},\mathcal {T};\mathbf {x}',\mathcal {T}')$ where $\beta \ll 1$ and $w_{\mathrm{lat}}$ satisfies the coaxiality requirement plus the fact that lateral fibers should preferentially connect neurons associated with the same or similar features. This point has been discussed in [21], where a general form for this function, with an additional small parameter measuring the degree of anisotropy in $w_{\mathrm{lat}}$, was proposed.

#### Remark 1

Suppose that $\mathcal {T}$ has eigenvalues $\lambda _{1}\gg \lambda _{2}\approx 0$. Then by (12) $\mathcal {T}$ is well approximated by the symmetric semi-definite tensor $\lambda _{1} \,{}^{t}\mathbf {e}_{1}\mathbf {e}_{1}$, and the set of these tensors is isomorphic to $\mathbb {R}^{+}_{\ast }\times \mathbb {P}^{1}$ where $\mathbb {P}^{1}$ is the real projective line. This shows how the ring model is embedded in the structure tensor model. This correspondence was discussed in [22]. This also comes back to taking the limit $\sigma _{2}=0$ in (11), that is, to considering the limit of very small image scale. The isometry group acting on this reduced model is $\mathbb {R}^{+}_{\ast }\times O(2)$. Despite the formal similarity between this formulation and the model which was considered in [43] to take account of the scale, the two are different. Here the $\mathbb {R}^{+}_{\ast }$ component refers to a measure of local contrast rather than scale.

In the rest of this paper we forget the spatial extension of V1 and concentrate on the structure tensor model in a single hypercolumn. The Wilson–Cowan equation therefore reads

14 $$ \frac{\partial v}{\partial t}(\mathcal {T},t)=(\mathcal {T},t) + \int _{SPD(2)} w\bigl(\mathcal {T};\mathcal {T}' \bigr)S_{\mu }\bigl(v\bigl(\mathcal {T}',t\bigr)\bigr)\,d\mathcal {T}' + I_{\mathrm{ext}}, $$

where $d\mathcal {T}$ is the volume element for the Riemannian metric $g_{\mathcal {T}}$, which we shall explicit in the next section, and $w(\mathcal {T};\mathcal {T}')$ depends only on the distance in $SPD(2)$. Our aim is now to describe the bifurcation of patterns from a homogeneous state of rest, thereby extending the orientation tuning analysis of Sect. 3.3.

### The Poincaré disc formulation of Eq. (14)

**Figure 6:**
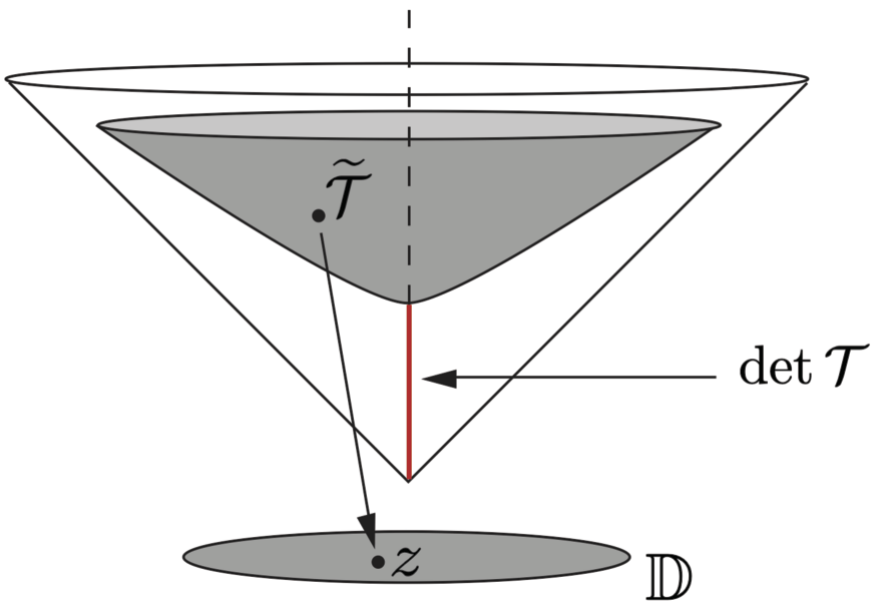
The hyperboloid $SSPD(2)$ and projection on the disc $\mathbb {D}$

In [12] Eq. (14) was reformulated in a particularly convenient form. First remark that any matrix in $SPD(2)$ can be written as $\mathcal {T}=\Delta \widetilde{\mathcal {T}}$, where $\Delta =\sqrt{\det \mathcal {T}}>0$ and therefore $\det \widetilde{\mathcal {T}}=1$. The subspace $SSPD(2)=\{\mathcal {T}/ \det \mathcal {T}=1\}$ is a hyperboloid which we further identify with the disc $\mathbb {D}=\{z\in \mathbb {C}/ |z|<1\}$ by the stereographic projection

$$ z=\frac{a-b+2ic}{2+a+b} $$

(see Fig. 6), where we have noted $\tilde{T}=(\begin{array}{cc} a & c\\ c & b \end{array})$. The metric induced by $SPD(2)$ on $SSPD(2)$ is the Minkowski metric of the hyperbolic surface of constant curvature +1, and the identification with $\mathbb {D}$ is the Poincaré disc of metric $g_{z}(dz,d\bar{z})=4(1-|z|^{2})^{-2}\, dz\, d \bar{z}$. Then the following proposition holds.

#### Proposition 1

*As a Riemannian manifold with metric* $g_{\mathcal {T}}$, $SPD(2) \simeq \mathbb {R}^{+}_{\ast }\times \mathbb {D}$. *The distance in these coordinates is expressed as follows*:

15 $$ d_{SPD(2)}\bigl(\Delta ,z;\Delta ',z' \bigr)=\sqrt{2\log { \biggl(\frac{ \Delta }{\Delta '} \biggr)}^{2}+2d_{\mathbb {D}}\bigl(z,z'\bigr)^{2}}, $$

*where* $d_{\mathbb {D}}(z,z')=2\operatorname{atanh}|(z-z')(1-\bar{z}z')^{-2}|$*is the distance in* $\mathbb {D}$. *The volume element is*

16 $$ d\mathcal {T}=2\sqrt{2}\frac{d\Delta }{\Delta }\,dm\bigl(z' \bigr), $$

*where* $dm(z)=4(1-|z|^{2})^{-1}\,dz\wedge d\bar{z}$*is the surface element in* $\mathbb {D}$.

We can now reformulate Eq. (14):

17 $$ \frac{\partial v}{\partial t}(\Delta ,z,t)=(\Delta ,z,t) + \int _{\mathbb {R}_{+}^{*}} \int _{\mathbb {D}}w\bigl(\Delta ,z;\Delta ',z' \bigr)S_{\mu }\bigl(v\bigl( \Delta ',z',t \bigr)\bigr)\frac{d\Delta '}{\Delta '}\,dm\bigl(z'\bigr) + I_{ \mathrm{ext}}. $$

The coefficient $2\sqrt{2}$ in (16) has been incorporated in *w*, which now is a function of $d_{SPD(2)}$ (Eq. (15)).

The existence and unicity of solutions of (17) was studied in [22], which gave similar results to the classical analysis of Wilson–Cowan equations in the Euclidean setting. As in Sect. 3.3 we concentrate on the problem of spontaneous tuning when $I_{\mathrm{ext}}=0$.

#### Remark 2

The identification of $SPD(2)$ with $\mathbb {R}^{+}_{\ast } \times \mathbb {D}$ is mathematically convenient as we shall see in the next section, but it can also be interpreted in the context of image processing by V1. It was noted in [21] that the modulus of $z\in \mathbb {D}$ is closely related to the distance from the pinwheel center in the hypercolumn. Indeed it has been measured experimentally that near pinwheel centers (at which all orientations are equally detected) the strength of orientation tuning is low, while the tuning curve response is much sharper (high selectivity) “far” from the pinwheel [38]. Now recall the coherence $\kappa =\frac{ \lambda _{1}-\lambda _{2}}{\lambda _{1}+\lambda _{2}}$, which measures the selectivity of a structure tensor with eigenvalues $\lambda _{1}$ and $\lambda _{2}$ (Sect. 4). Expressed in *z* coordinates for a structure tensor in $SSPD(2)$, it gives $\kappa =\frac{2|z|}{1+|z|^{2}}$. Hence $z=0$ corresponds to the pinwheel (no orientation), whereas $z=|z|e^{i\theta }$ close to the boundary of $\mathbb {D}$ corresponds to a point in the hypercolumn with high selectivity in the orientation *θ*. About the Δ coordinate, it can be noted that the difference between images seen at a given scale with structure tensors with same component $z\in \mathbb {D}$ and determinants Δ and $\Delta '$ relates just to a difference of contrast between the two at scale $\sigma _{2}$.

Before considering the bifurcation problem for (17), we need to recall some important facts about the geometry of $\mathbb {D}$ and in particular its isometries.

### Isometries of the Poincaré disc

The direct isometries (preserving the orientation) in $\mathbb {D}$ are the elements of the special unitary group, denoted ${\operatorname{SU}}(1,1)$, which are the matrices

$$ \begin{bmatrix} \alpha & \beta \\ \overline{\beta }& \overline{\alpha } \end{bmatrix}\quad \text{such that } \vert \alpha \vert ^{2}- \vert \beta \vert ^{2}=1. $$

These elements act in $\mathbb {D}$ by

18 $$ \gamma \cdot z = \frac{\alpha z+\beta }{\overline{\beta }z+\overline{ \alpha }},\quad z\in \mathbb {D}. $$

Let $\alpha =\alpha _{1}+i \alpha _{2}$, $\beta =\beta _{1}+i \beta _{2}$ in (18). The corresponding isometry in the space of structure tensors is given by

19 $$ \tilde{\gamma } \cdot \mathcal{T}=\,{}^{t} \tilde{ \gamma } \mathcal{T} \tilde{\gamma }, $$

where

20 $$ \tilde{\gamma }= \begin{bmatrix} \alpha _{1}+\beta _{1} & \alpha _{2}+\beta _{2} \\ \beta _{2}-\alpha _{2} & \alpha _{1}-\beta _{1} \end{bmatrix} \in \operatorname{SL}(2,\mathbb {R}). $$

Because isometries are conformal maps, they preserve angles. However, they do not transform straight lines into straight lines. Given two points $z\neq z'$ in $\mathbb {D}$, there is a unique geodesic passing through them, which is the arc of circle containing *z* and $z'$ and intersecting the unit circle at right angles. This circle degenerates to a straight line when the two points lie on the same diameter. Any geodesic uniquely defines a reflection through it. Reflections are orientation reversing, one representative is the complex conjugation *κ* (reflection through the geodesic $\mathbb {R}$): $\kappa \cdot z= \overline{z}$. The full symmetry group of the Poincaré disc is therefore

21 $$ \mathrm{U}(1,1)=\operatorname{SU}(1,1)\cup \kappa \cdot \operatorname{SU}(1,1). $$

A fundamental property of $\operatorname{SU}(1,1)$ is that any element is the product of three “simple” transformations belonging respectively to three one-parameter subgroups *K*, *A*, and *N*, the definition of which is given below. Hence one can write $\operatorname{SU}(1,1)= {KAN}$ (Iwasawa decomposition, see [32]). The corresponding subgroups of $SSPD(2)$ can be computed using (20).

#### Definition 1

$$\left\{ \begin{array}{c} K=\{r_{\varphi }=[\begin{array}{cc} e^{i\varphi /2} & 0\\ 0 & e^{-i\varphi /2} \end{array}],\varphi \in S^{1}\},\\ A=\{a_{\tau }=[\begin{array}{cc} \cosh\frac{\tau }{2} & \sinh\frac{\tau }{2}\\ \sinh\frac{\tau }{2} & \cosh\frac{\tau }{2} \end{array}],\frac{\tau }{2}\in R\},\\ N=\{n_{s}=[\begin{array}{cc} 1+is & -is\\ is & 1-is \end{array}],s\in R\}. \end{array}\right.$$

**Figure 7:**
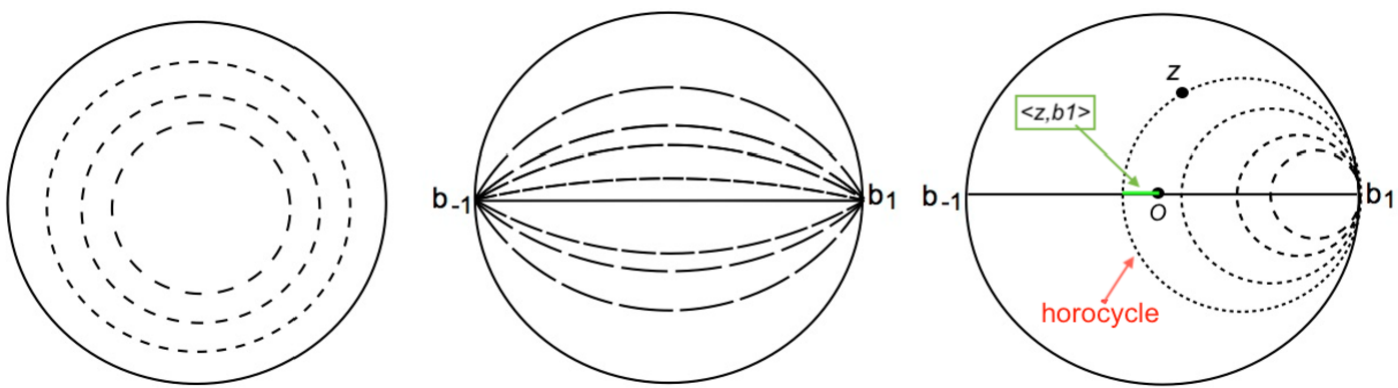
The orbits of the subgroups *K*, *A*, *N*. The green segment on the right panel illustrates the “inner product” $\langle z,b \rangle $ when $b=b_{1}$ (see Sect. 5.1)

We describe the action of these three groups, see Fig. 7 for an illustration.

- *K* is isomorphic to $SO(2)$: $r_{\varphi }\cdot z=e ^{i\varphi } z$ for $z\in \mathbb {D}$. Its orbits are concentric circles. In $SSPD(2)$ it acts by rotating with an angle $\varphi /2$ the orthonormal basis $(\mathbf{e}_{1},\mathbf{e}_{2})$ in which the coordinates of $\mathcal{T}$ are expressed.
- The elements of *A* are sometimes called “boosts” in the theoretical physics literature [1]. All orbits converge to the limit points $b_{\pm 1}=\pm 1$ on the unit circle (boundary of the Poincaré disc). In particular the diameter $b_{1}b_{-1}$ is an orbit. The action of $a_{\tau }\in A$ on $SSPD(2)$ consists in scaling the first vector of the orthonormal basis $(\mathbf{e}_{1},\mathbf{e}_{2})$ by $e^{\tau }$ and the second one by $e^{-\tau }$.
- The orbits of *N* are the circles tangent to the unit circle at the point $b_{1}$. These orbits are called *horocycles*. There is no obvious interpretation of these transformations in the space of structure tensors.

Any nontrivial direct isometry falls into one of the following classes (representatives of which are given by elements of *K*, *A*, and *N* respectively):

(i) Elliptic elements: one fixed point in $\mathbb {D}$;
(ii) Hyperbolic elements: two (and only two) fixed points on the unit circle;
(iii) Parabolic elements: one and only one fixed point on the unit circle.

## Tuning of textures in $\mathbb {D}$

The aim of this section is to provide the tools which are necessary to study the bifurcation of solutions of (17) when $I_{\mathrm{ext}}=0$ and to describe (without proofs) the main results which were obtained in [12, 13]. Compared to previous models in which the domain *Ξ* was Euclidean or spherical, the novelty here is that $\varXi =\mathbb {R}^{+}_{\ast }\times \mathbb {D}$ and $\mathbb {D}$ is equipped with hyperbolic geometry. Fourier analysis is more involved than in $\mathbb {R}^{2}$ or $S^{2}$ and is shortly described in Sect. 5.1.

Since the $\mathbb {R}^{+}_{\ast }$ part of the domain does not pose a new challenge, we shall for the sake of simplicity consider solutions which are independent of the variable Δ. Then Eq. (17) reduces to

22 $$ \frac{\partial v}{\partial t}(z,t)=-v(z,t) + \int _{\mathbb {D}} w\bigl(z;z'\bigr)S_{ \mu } \bigl(v\bigl(z',t\bigr)\bigr)\,dm\bigl(z'\bigr), $$

and $w(z;z')$ is a function of distance $d_{\mathbb {D}}(z;z')$ only: $w(z,z')=h(d_{\mathbb {D}}(z,z'))$, where *h* is the function defined in (7). The integral part of this equation can be written in a convolution form as follows.

Let *O* be the center of $\mathbb {D}$ and *dg* represent the Harr measure on the group $G=\operatorname{SU}(1,1)$ (see [30]), normalized by

$$ \int _{G} f(g\cdot O)\,dg= \int _{\mathbb {D}}f(z)\,dm(z) $$

for all functions in $L^{1}(\mathbb {D})$.

### Definition 2

Given $f_{1}$, $f_{2}$ in $L^{1}(\mathbb {D})$, we define their convolution by

$$ (f_{1}\ast f_{2}) (z)= \int _{G}f_{1}(g\cdot O)f_{2} \bigl(g^{-1}\cdot z\bigr)\,dg. $$

### Proposition 2

*Let* $W(z)=w(z,O)$, *then for all bounded functionvin* $\mathbb {D}$*the following equality holds*:

$$ \int _{\mathbb {D}} w\bigl(z;z'\bigr)S_{\mu } \bigl(v\bigl(z',t\bigr)\bigr)\,dm\bigl(z'\bigr)=W \ast S_{\mu }(v) (z). $$

### Proof

This is a consequence of the *G*-invariance of *w*: $w(g\cdot z,g \cdot z')=w(z,z')$. Writing $z'=g\cdot O$ and integrating over *G* instead of $\mathbb {D}$, the result follows easily. □

### Spectral and Fourier analysis in $\mathbb {D}$

Classical Fourier analysis does not apply in $\mathbb {D}$. Nevertheless a similar theory exists, however with notable differences. In the following only some basic notions are exposed, and I refer to [30] for a thorough exposition and to [1] for a theoretical physics point of view.

Given a point $z\in \mathbb {D}$, there is a unique couple of hyperbolic and parabolic orbits which intersect at *z*. Hence we may write $z=n_{s} a_{\tau }\cdot O$, $(s,\tau )\in \mathbb {R}^{2}$ and *O* the center of the disc. The values *s* and *τ* are the *horocyclic coordinates* of *z*.

Now let $b\in \partial \mathbb {D}$ be a point on the unit circle. We define the “inner product” $\langle z,b\rangle $ as the smallest signed distance from *O* to the horocycle based at *b* and passing by *z*, see the picture on the right of Fig. 7. This value is clearly invariant under rotations in *K*. By a suitable rotation, we take $b=b_{1}=1$, then by (18) and the expression of the distance $d_{\mathbb {D}}$ (see Proposition 1), one has $\langle z,b_{1}\rangle =\tau $. Note that $\langle z,b\rangle $ is also invariant by the action of the parabolic group *N*. Finally, elementary calculus shows that in horocyclic coordinates the surface element is $dm(z)=e^{-\tau }\,ds\,d\tau $.

Let $\Delta _{\mathbb {D}}$ be the Laplace–Beltrami operator in $\mathbb {D}$, which in Cartesian coordinates $z=x+iy$ reads

$$ \Delta _{\mathbb {D}}= \frac{(1-x^{2}-y^{2})^{2}}{4}\bigl(\partial ^{2}_{x}+\partial ^{2}_{y} \bigr). $$

By construction this operator is invariant under the transformation group $\mathrm{U}(1,1)$.

#### Proposition 3

*The functions*

23 $$ e_{\rho ,b}(z)=e^{(i\rho +\frac{1}{2})\langle z,b \rangle }, \quad \rho \in \mathbb {C}, $$

*are eigenfunctions of* $\Delta _{\mathbb {D}}$*with eigenvalues* $-(\rho ^{2}+ \frac{1}{4})$.

Real eigenvalues of $\Delta _{\mathbb {D}}$ correspond to taking *ρ* real or purely imaginary in (23). The latter case is irrelevant for us as it corresponds to exponentially diverging eigenfunctions when $|z|\rightarrow 1$. Therefore the real spectrum of $-\Delta _{\mathbb {D}}$ is continuous and bounded from below by $1/4$. The corresponding elementary eigenfunctions are the analogue of the Euclidean plane waves. When $b=b_{1}$, they are expressed as $e_{\rho ,b}(z)=e^{(i\rho +1/2)\tau }$ with *τ* being the spatial coordinate of the propagation. These waves propagate along the geodesics emanating from $b_{1}$, which cross orthogonally the horocycles. The point $b_{1}$ is the “source”, which makes a big difference with Euclidean case where there is no preferred direction (reflectional symmetry). Because of the factor $e^{\tau /2}$, these plane waves are not periodic. However, this term balances the growth of the surface element in the energy density, which in horocyclic coordinates is $|e_{\rho ,b}(s,\tau )|^{2}e^{-\tau }\,ds\,d\tau $.

It follows from Helgason’s theory that any eigenfunction of $\Delta _{\mathbb {D}}$ can be expressed as an integral over a distribution of the elements $b\in \partial \mathbb {D}$:

#### Theorem 2

*Any eigenfunction of the operator* $\Delta _{\mathbb {D}}$*admits a decomposition of the form*

$$ \int _{\partial D}{e_{\rho ,b}(z)\, dT_{\rho }(b)}, $$

*where* $T_{\rho }$*is a distribution defined on the unit circle∂D*.

#### Definition 3

Given a function *f* on $\mathbb {D}$, its *Helgason–Fourier transform* is defined by

24 $$ \tilde{f}(\rho , b) = \int _{\mathbb {D}}{f(z)e_{-\rho ,b}(z) \,dm(z)} $$

for all $\rho \in \mathbb {C}$, $b\in \partial \mathbb {D}$ for which the integral exists.

#### Theorem 3

(Inverse Helgason–Fourier transform)

*Forfa differentiable function with compact support in* $\mathbb {D}$, *the following formula holds*:

25 $$ f(z) = \frac{1}{2\pi } \int _{\mathbb {R}}\int _{\partial \mathbb {D}}{\tilde{f}( \rho ,b))e_{\rho ,b}(z)\rho \tanh (\pi \rho ) \,d\rho \,db}, $$

*wheredbis normalized by* $\int _{\partial \mathbb {D}}db=1$.

The following proposition is useful when dealing with integral equations in $\mathbb {D}$.

#### Proposition 4

*Let* $f_{1},f_{2}\in L^{1}(\mathbb {D})$*and suppose that* $f_{2}$*is invariant under the rotation group K*. *Then* $\widetilde{f_{1}\ast f_{2}}= \tilde{f}_{1}\tilde{f}_{2}$, *wheref̃indicates the Helgason–Fourier transform off*.

There is another way to express the eigenfunctions of the Laplace–Beltrami operator, by choosing, instead of the horocyclic coordinates, the *geodesic coordinates* $(\tau ,\theta )$ such that $z=\tanh (\tau /2)e^{i\theta }$, $0\leq \tau <+\infty $, $\theta \in \mathbb {S}^{1}$. This will be useful when looking for states in a bounded circular subdomain of $\mathbb {D}$ in Sect. 5.2.3. An arbitrary eigenfunction can be expanded as

26 $$ \varPsi _{l}(z)=\sum_{-\infty }^{+\infty }a_{m} \mathcal {P}^{m}_{l}\bigl(\cosh (\tau )\bigr)e ^{im\theta }, $$

where $\mathcal {P}^{m}_{l}$ are associated Legendre polynomials, $a_{m} \in \mathbb {C}$ and $l=-\frac{1}{2}\pm i\rho $ [1].

### Bifurcation of patterns in $\mathbb {D}$

As in the Euclidean case (Sect. 3.4), the hyperbolic domain $\mathbb {D}$ is not compact and the spectrum of $\Delta _{\mathbb {D}}$ is continuous. Moreover, the eigenvalues have in general infinite multiplicity because they are independent of the direction $b\in \partial \mathbb {D}$. In order to compute bifurcated branches of solutions of (22), one would like to apply the same idea as in Sect. 3.4, that is, to look for *periodic patterns*. Periodic patterns in $\mathbb {D}$ are functions invariant under the action of a discrete subgroup *Γ* of transformations in $G=\mathrm{U}(1,1)$. Thanks to the *G*-invariance of (22), one can project this equation on the quotient domain $\mathbb {D}/\varGamma $. If this domain happens to be compact, which is not always the case, the restricted spectrum will be discrete with eigenvalues of finite multiplicity, so that the method of Sect. 3.2 applies.

In Sect. 5.2.1 a simple case of bifurcation of periodic patterns, which is the hyperbolic counter part of the bifurcation of stripes in Turing-like instability, is presented. These periodic waves are traveling at constant speed, while in the Euclidean case they are stationary. Unfortunately, they do not appear to be physically relevant because of their energy density growth to infinity towards the boundary of $\mathbb {D}$.

The case of “true” periodic patterns was discussed in [12, 13, 20], with detailed computations when the fundamental domain of the pattern is a regular octagon. This is presented in Sect. 5.2.2.

Finally I also present briefly in Sect. 5.2.3 the simpler case of a compact subdomain inside $\mathbb {D}$ with prescribed boundary conditions, which gets rid of the high degeneracy induced by the $\operatorname{SU}(1,1)$ invariance of the problem. It can indeed be argued that since natural images can only produce a bounded set of structure tensors, it is relevant to take for the domain a disc in $\mathbb {D}$, invariant by the action of the rotation group $K\simeq SO(2)$, in order to incorporate the ring model in this hyperbolic model. From the group theoretic point of view the analysis is then similar to the ring model and the bifurcated branches of solutions can be computed analytically. Compared to the ring model, the additional information lies in the “radial” modes of the critical eigenvalue problem.

Let us mention for completeness the bifurcation of localized solutions (bumps) in [24]. Localized solutions decay to 0 as $z\rightarrow \infty $. In the case of a planar domain such states are known to exist and play an important role in cortex modeling, see [17] and the references therein. No general method exists to compute these solutions, and further restrictions, both on the equation and on the type of solutions, are needed. Already in the Euclidean case the technical difficulties are quite involved. Roughly speaking the method consists in considering the radial variable as a time variable and treating the equation as a dynamical system, which requires to choose the connectivity function such that it is the kernel of a differential operator (this idea was already applied by [35] to numerically compute localized solutions in a planar domain). I refer to [23, 24] for further details.

#### Periodic hyperbolic waves

**Figure 8:**
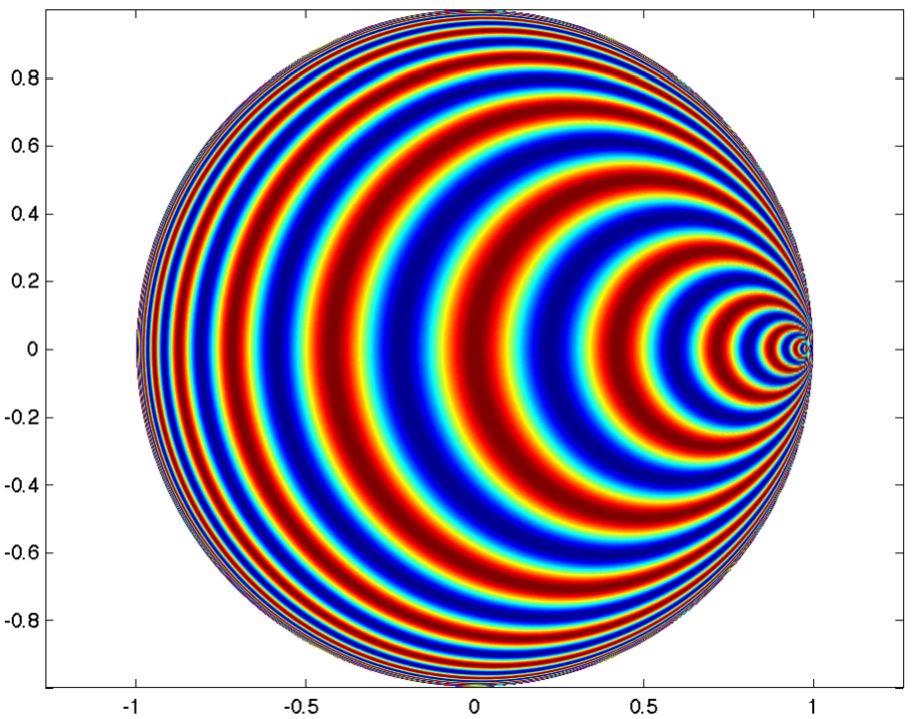
An example of hyperbolic waves with source at $b_{1}$

The elementary waves $e_{\rho ,b}(z)=e^{(i\rho +1/2)\langle z,b\rangle }$ are not periodic except when $\rho =\alpha +i/2$, $\alpha \in \mathbb {R}$. Indeed, in horocyclic coordinates, if we take $b=b_{1}$ (see Fig. 7), then $e_{\rho ,b_{1}}(n_{s}a_{\tau }\cdot O)=e ^{i\alpha \tau }$. Figure 8 illustrates how these waves look like. Any point on the unit circle is a source of similar waves rotated by the corresponding angle. Suppose that we look for solutions of (22) which have the same properties as these periodic waves: (i) invariance under the action of the horocyclic group *N*, (ii) invariance by a discrete subgroup of the one-parameter group *A*: $v((\tau +k)\cdot O)=v( \tau \cdot O)$, all $\tau \in \mathbb {R}$, $k\in \mathbb {R}$ fixed (the “period”). By periodicity the action of *A* in the space of such functions projects onto the 1-torus $\mathbb {R}/k\mathbb {Z}\simeq \mathbb {S}^{1}$, which is compact.

By condition (i) Eq. (22) becomes

$$\begin{aligned} \partial _{t} v =&-v(t,\tau )+ \int _{\mathbb {R}}\int _{\mathbb {R}}w(n_{s}a_{\tau } \cdot O,n_{s}a_{\tau }\cdot O)S(v(t,a_{\tau '}) \,ds'e^{-\tau '}\,d\tau ' \\ =&-v(t,\tau )+ \int _{mR}{ \biggl( \int _{\mathbb {R}}w(a_{\tau -\tau '}\cdot O,n_{s} \cdot O)\,ds \biggr)}S\bigl(v\bigl(t,\tau '\bigr)\bigr)\,d\tau ', \end{aligned}$$

where for the second equality we have used the formula $a_{\tau }n _{s}=n_{se^{\tau }}a_{\tau }$ (see [30]). The integral in parenthesis is a function $\chi (\tau -\tau ')$, so that we may rewrite the equation with the usual convolution product

27 $$ \partial _{t} v=-v+\chi \ast S(v), $$

where now $v=v(t,\tau )$, $\tau \in \mathbb {R}$, and the equation is invariant by space-time translations $(t,\tau )\mapsto (t-t_{0},\tau -\tau _{0})$.

**Figure 9:**
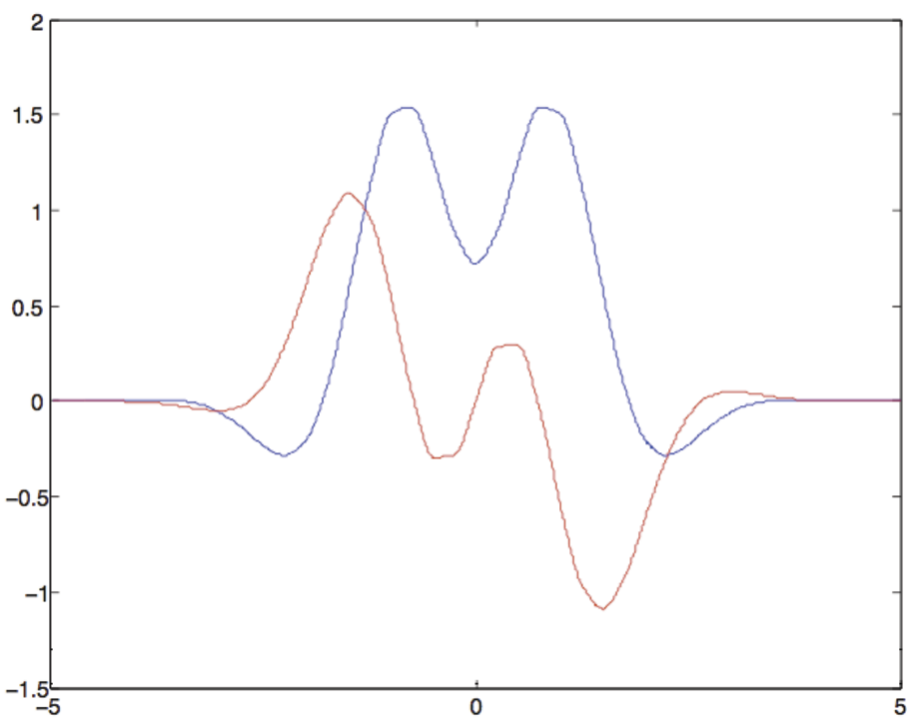
Real (blue) and imaginary (red) parts of *χ̃* (with $\sigma _{1}=0.9$, $\sigma _{2}=1$, $A=0.6$ in (7)) for periodic waves

We now look for the bifurcation of periodic hyperbolic waves from a basic state using the method of Sect. 3.2. By definition, basic state solutions $v(\mu )$ of (27) are independent of time and are translation invariant in *τ*, hence constants. By a suitable change of variable, we can set wlog $v_{\mu }=0$ and $S'_{\mu }(0)=\mu $. The bifurcation point is obtained by solving the eigenvalue equation $\lambda u=-u+\mu \chi \ast u$ with $u(\tau )=e^{i\alpha \tau }$, $\alpha \in \mathbb {R}$. In Fourier space it gives $\lambda (\alpha )=-1+\mu \tilde{\chi }(\alpha )$, where *χ̃* is the Fourier transform of *χ* in the usual sense. Numerical computation shows that *χ̃* has real and imaginary parts varying with *α* as in Fig. 9 for a typical “Mexican hat” function $w(z,z')=h(d_{\mathbb {D}}(z,z'))$. The most unstable eigenvalues correspond to the maximum of the real part of *χ̂*, which in Fig. 9 occurs at $|\alpha |=\alpha _{c}\approx 0.76$. The critical value $\mu _{0}$ of the bifurcation parameter is the value at which the real part of $\lambda (\alpha _{c})$ vanishes and the corresponding eigenvalues are simple and purely imaginary: $\pm i\omega _{0}$ (in the example $\mu _{c}\approx 0.65$ and $\omega _{0}\approx 0.04$). This is Hopf bifurcation; however, it reduces to steady-state bifurcation thanks to space and time translational invariance. Indeed let us look for solutions in the form $v(t,\tau )=\tilde{v}(t,\eta t+\tau )$, *η* being an unknown speed of propagation of waves. In the new variable $s=\eta t-\tau $, Eq. (27) reads

28 $$ \partial _{t} \tilde{v} = -\eta \partial _{s}\tilde{v} -\tilde{v} + \chi \ast S_{\mu }(\tilde{v}). $$

The above linear analysis shows that bifurcation occurs for this equation with a 0 double eigenvalue at $\mu _{0}$, $\eta =\eta _{c}= \omega _{0}/\alpha _{c}$ and critical modes $\zeta =e^{i\alpha _{c} s}=e ^{ i(\alpha _{c}\tau +\omega _{0}t)}$ and *ζ̄*. Moreover, since we are now looking for solutions with $2\pi /\alpha _{c}$ periodicity in *s*, the symmetry group is $\mathbb {R}/\alpha _{c}\mathbb {Z}\simeq SO(2)$, which is compact. Therefore the center manifold reduction theorem applies. This is now a steady-state bifurcation with a double 0 eigenvalue as a consequence of the $SO(2)$ invariance of *ṽ*. Hence $\dim V_{0}=2$, however there is an additional variable *η*. Let us write points in $V_{0}$ as $r[e^{i\varphi }\zeta + e^{-i\varphi }\bar{\zeta }]$, then the real and imaginary parts of the bifurcation equation for stationary solutions read as follows (as a consequence of the $SO(2)$ invariance, see [14]):

$$\begin{aligned}& 0= rg\bigl(\mu ,r^{2}\bigr), \\& 0= -\eta \alpha _{c} + \omega _{0} + h\bigl(\mu ,r^{2}\bigr), \end{aligned}$$

where *g* and *h* are real functions of order at least one in $|\mu |+r^{2}$ [14]. The pase *φ* is arbitrary. The second equation solves for $\eta =\eta _{c}+o(1)$ and the first one gives pitchfork branches of solutions $r=0$ (basic state) and $r=\pm [\sqrt{c\mu }+O(\mu )]$ (if $c=0$ one must go to higher order). Coming back to (27), the solutions have the form $v=v_{\mu }(\eta t+\tau )$. The time dependance corresponds to a uniform speed of translation along the “space” variable *τ* (traveling waves), and this speed tends to $\eta _{c}$ as *μ* tends to 0. It is interesting to note that these solutions are the hyperbolic version of the “rolls” or “stripes” solutions which are well known in physics, like convection rolls in fluids, stripes in reaction-diffusion systems. There is, however, a notable difference: in our case the solutions depend on time and are traveling at constant speed from a “source” situated on the boundary of the Poincaré disc. The occurrence of stationary waves is non-generic. This is because in hyperbolic geometry these waves have a preferred direction given by the “source” on $\partial \mathbb {D}$.

#### Periodic patterns

The bifurcation the periodic waves in the previous section has the advantage of simplicity; however, their density of energy is not bounded in $\mathbb {D}$ as it grows like $e^{-\tau }\,ds\,d\tau $, which does not look physically meaningful. This drawback would disappear with solutions which are periodic in $\mathbb {D}$, that is, which are invariant under the action of a discrete subgroup of $\operatorname{SU}(1,1)$ with a compact fundamental domain (or tile). Subgroups sharing these conditions are called *cocompact Fuchsian groups*. Their study was initially developed by Poincaré (who gave the name “Fuchsian group”), see [33]. We quickly review some important general features of these groups before passing to a specific example. Let *Γ* be a cocompact Fuchsian group. Its *fundamental domain* is a compact set $F_{\varGamma }\subset \mathbb {D}$ such that

(i) if $\gamma \neq id\in \varGamma $, then $\gamma F_{\varGamma } \cap \mathring{F_{\gamma }}$;
(ii) $\bigcup_{\gamma \in \varGamma }\gamma F_{\varGamma }=\mathbb {D}$.

The fundamental domain of a cocompact Fuchsian group can always be built as a (hyperbolic) polygon, called Dirichlet region [33].

A cocompact Fuchsian group contains no parabolic element, that is, no element conjugates to an element in the parabolic subgroup *N*. When it does not contain elliptic elements (conjugating to a rotation in *K*) as well, we call this group a *lattice group* of $\mathbb {D}$. The action of a lattice group *Γ* contains no fixed point in $\mathbb {D}$. Therefore the quotient surface $\mathbb {D}/\varGamma $ is a compact Riemann surface, which can be seen as the fundamental domain with “opposite” sides being identified by periodicity. Conversely, a compact Riemann surface is the fundamental domain of a lattice group of $\mathbb {D}$ if and only if it has genus $g\geq 2$. The case $g=1$ corresponds to a lattice in the Euclidean plane, for which the quotient $\mathbb {R}^{2}/\varGamma $ is a torus. Lattice groups are classified according to the group $G_{\varGamma }$ of automorphisms of $\mathbb {D}/\varGamma \simeq F_{\varGamma }$. In the Euclidean case $G_{\varGamma }=H\ltimes \mathbb {R}^{2}/\mathbb {Z}^{2}$, where *H* is the holohedry of the lattice and there are only four holohedries. By contrast, in the hyperbolic case there are infinitely many groups $G_{\varGamma }$, however they always have finite order.

Coming back to Eq. (22), suppose that we look for solutions which are invariant under a lattice group *Γ*. Since we assume that the connectivity function depends only on the distance $d_{\mathbb {D}}(z,z')$, the equation is invariant under the action of $\operatorname{SU}(1,1)$, and therefore we can reduce the analysis to the compact quotient domain $\mathbb {D}/\varGamma $:

29 $$ \frac{\partial v}{\partial t}(z,t)=(z,t) + \int _{\mathbb {D}/\varGamma } w\bigl(z;z'\bigr)\,dm \bigl(z'\bigr). $$

It follows that the spectrum of the linearized operator is discrete, the eigenvalues have finite multiplicities and (29) is invariant under the action of $G_{\varGamma }$. Therefore one can apply equivariant bifurcation theory to compute the solution branches and classify them according to their isotropy, as exposed in Sect. 3.2.

The leading part of the bifurcated solutions are eigenfunctions of the Laplace–Beltrami operator which satisfy the periodicity and isotropy conditions. They were named H-planforms in [12]. Unfortunately there are several drawbacks to this program. The first one is that the eigenvalues and eigenfunctions are difficult to compute, as there exists no explicit formula to express them. More precisely, for *Γ*-periodic eigenfunctions the distribution $T_{\rho }$ in Theorem 2 does not admit any explicit expression. Efficient numerical algorithms are necessary to compute these eigenvalues and the corresponding H-planforms. The second drawback is that unlike the Euclidean case the size of the fundamental domain is fixed by its type, which implies that the eigenvalues depend on $F_{\varGamma }$, hence on *Γ*. Whether a bifurcation of *Γ*-periodic states would correspond to a destabilization of the basic state under random perturbations, or whether another bifurcation with another lattice group would have already occurred, is not known.

**Figure 10:**
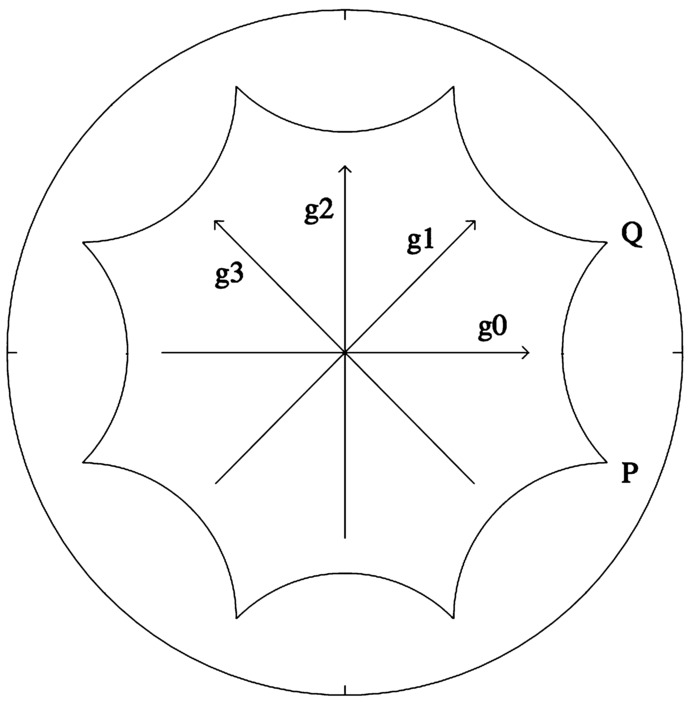
The fundamental octagonal domain $\mathcal {O}$ and its generators (redrawn from [1])

Nevertheless, a thorough analysis was performed by [13, 20] in the case when the fundamental domain is the regular octagon $\mathcal {O}$ shown in Fig. 10. To end this section, I will quickly review this study, which is also interesting for its own.

**Figure 11:**
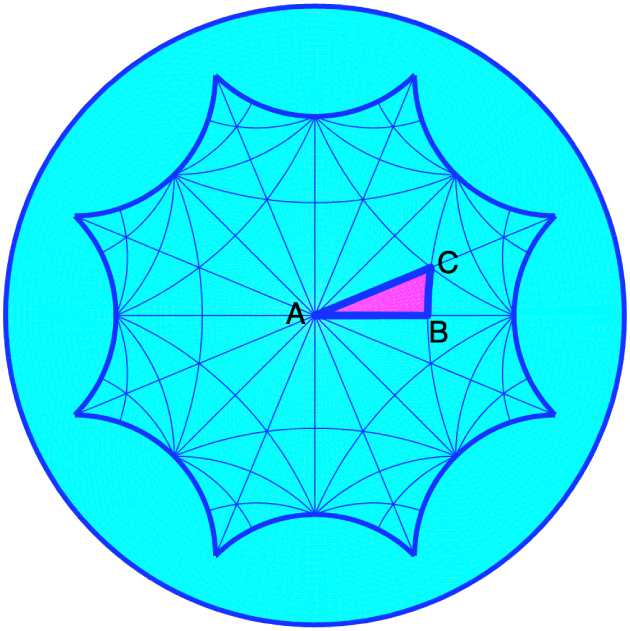
Tesselation of the regular hyperbolic octagon by successive reflections of the triangle colored in purple in the plot

The lattice group of $\mathcal {O}$ is generated by four hyperbolic translations

30 $$ g_{0} = \begin{pmatrix} 1+\sqrt{2} & \sqrt{2+2\sqrt{2}} \\ \sqrt{2+2\sqrt{2}} & 1+\sqrt{2} \end{pmatrix} ,\qquad g_{j} = r_{j\pi /4}g_{0}r_{-j\pi /4}\quad (j=1,2,3) $$

where $r_{\varphi }$ denotes the rotation of angle *φ* around the origin in $\mathbb {D}$. The opposite sides of the octagon are identified by periodicity, so that the corresponding quotient surface $\mathbb {D}/\varGamma $ is isomorphic to a “double doughnut” (genus $g=2$), which is the simplest case of periodic tiling [1]. The full symmetry group of this fundamental domain, including reflections, contains 96 elements and is denoted by $G^{*}$. It can be generated as follows. Let $\mathcal {T}$ be a hyperbolic triangle with angles $\pi /8$, $\pi /3$, and $\pi /2$ at the vertices *A*, *B*, and *C* respectively. Let *Λ* be the group generated by reflections through the edges of $\mathcal {T}$. It can be shown that $G^{*}\simeq \varGamma /\varLambda $. This means that $\mathcal {O}$ is tesselated by 96 copies of $\mathcal {T}$ as illustrated in Fig. 11. Furthermore, $G^{*}\simeq G\cup \kappa G$, where *G* is the group of direct isometries generated respectively by the rotation by $\pi /2$ around the vertex *B* and $\pi /3$ around the vertex *C* of $\mathcal {T}$. The element *κ* is the reflection through the real axis in $\mathbb {D}$. It can be shown that *G* is isomorphic to the group $GL(2,3)$ of invertible $2\times 2$ matrices over the field $\mathbb {Z}/3\mathbb {Z}$.

Once the group $G^{*}$ is known, its absolutely irreducible representations can be determined, which was done in [13] with the help of the computer algebra software programm GAP. A total of 27 different types of solutions have been identified with the equivariant branching lemma, and the corresponding *H*-planforms have been computed numerically. Table 1 gives a synthetic view of these results.

**Table 1:** The number of absolutely irreducible representations and the corresponding number of isotropy types of bifurcated solutions

Dimension of absolutely irred. representation	1	2	3	4
Number of nonequivalent representations	4	2	4	3
Number of isotropy types with $\dim V_{H}=1$	4	3	10	10

**Figure 12:**
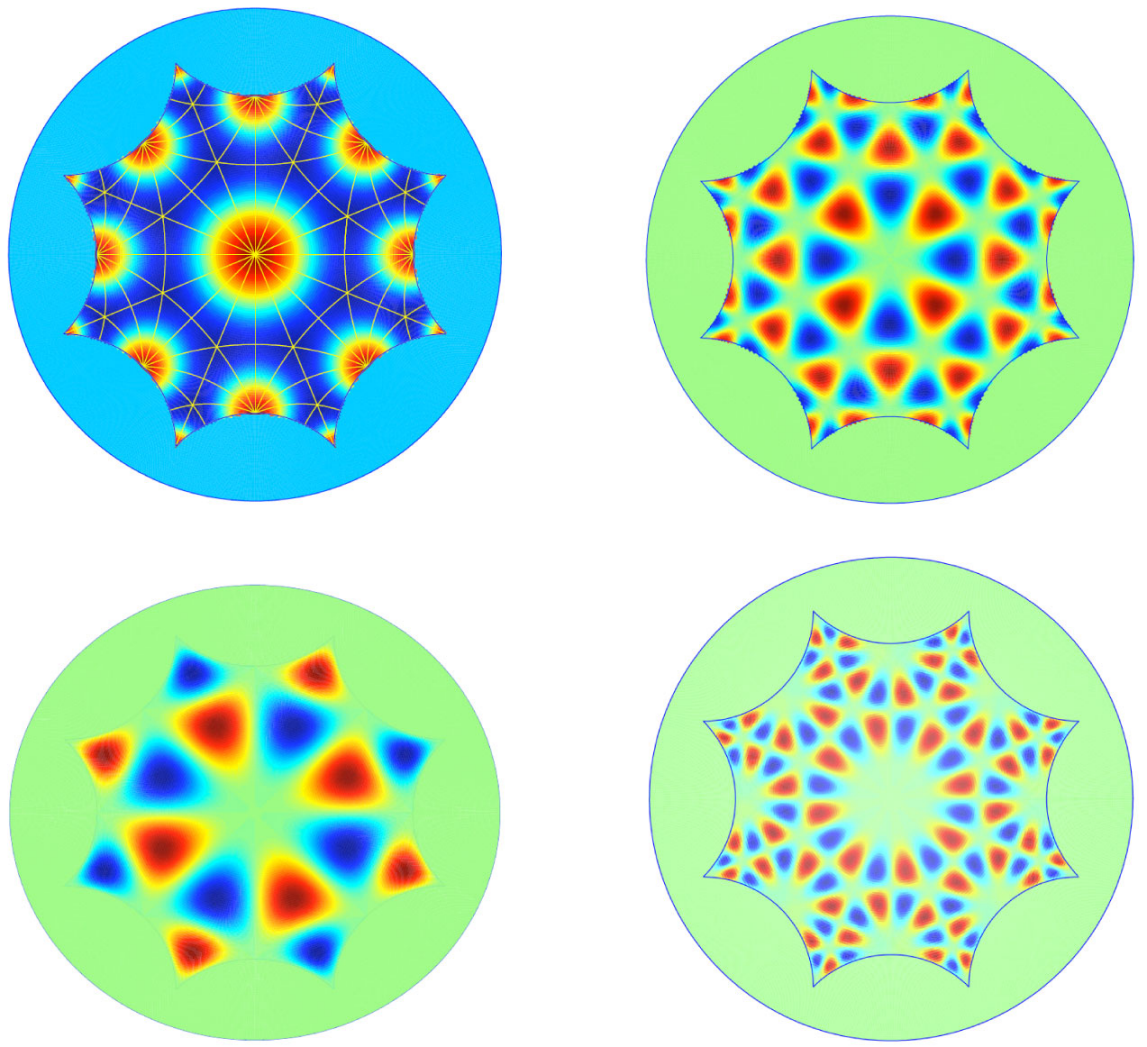
The four H-planforms associated with the four irreducible representations of dimension 1, see text. Upper left: H-planform with full $G^{*}$ symmetry. Lower right: H-planform with symmetry *G*

**Figure 13:**
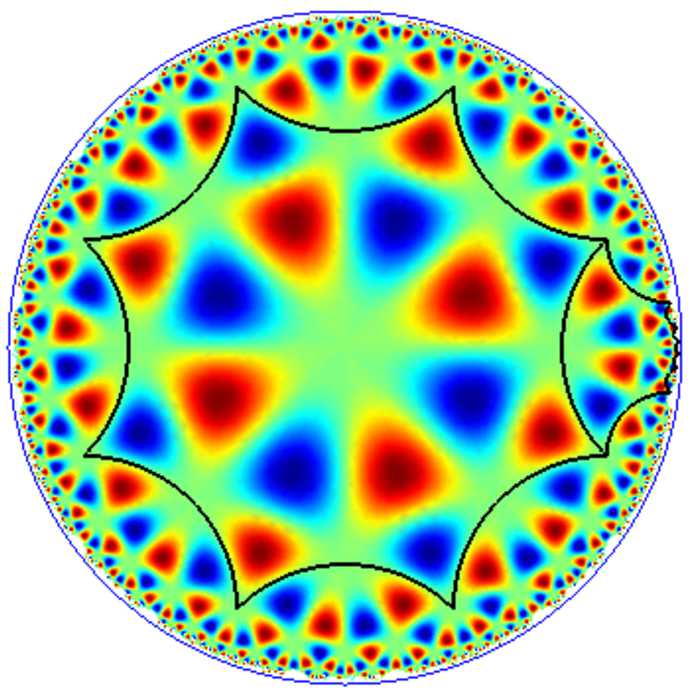
An example of tiling with an H-planform (see text). The polygon marked with black line edges on the right of $\mathcal {O}$ is the copy of this octagon by the hyperbolic translation $g_{0}$ (see (30))

Figure 12 shows four examples of such patterns, which correspond to the cases in which $\dim V_{0}=1$ (the four absolutely irreducible representations of dimension 1). The physical relevance of these patterns is an open question. Note that identifying the isotropies of an H-planform from its picture is an interesting exercise… For those in upper right and lower left pictures, the answer is in [13]. To end this section, Fig. 13 illustrates the tiling of $\mathbb {D}$ with the H-planform on the lower left corner of Fig. 12.

In the 4-dimensional representation spaces, solutions with submaximal isotropy (that is, with $\dim V_{H}=2$) do also exist, and in one case time-dependent solutions (related to the presence of heteroclinic cycles) can also bifurcate [20].

#### A simpler approach: restricting to a bounded domain in $\mathbb {D}$

This approach originates from the need to incorporate the structure tensor model in the spatially extended V1 in a tractable way [21]. A more direct method, starting from the octagonal-periodic patterns presented in the previous section, was also investigated, but this leads to substantially more complicated computations.

It sounds physically relevant to assume that the structure tensors that are detected by neurons in hypercolumns are bounded and therefore belong to some compact domain in $\mathbb {D}$. Since orientation is part of the features represented by the structure tensor, it is also relevant to assume that the domain is a disc. Let therefore $\varOmega \subset \mathbb {D}$ be the disc of radius $\tanh (\omega /2)<1$. We use geodesic coordinates $(\tau ,\theta )$ for points in *Ω*: $z=\tanh (\tau /2)e^{im \theta }$ (see the last paragraph of Sect. 5.1). Note that of all the isometries of $\mathbb {D}$ only rotations $r_{\varphi }$ around the center of the disc and reflections through its diameter remain. Hence when $\mathbb {D}$ is replaced by *Ω*, the symmetry group of Eq. (22) reduces to $O(2)$, which is the same symmetry group as in the ring model of Sect. 6. We also assume that the structure tensors vanish on the boundary of *Ω*.

The Wilson–Cowan equation now reads as follows:

31 $$ \begin{aligned}[b] \frac{\partial v}{\partial t} &=(\tau , \theta ,t)+ \int _{0}^{\omega } \int _{0}^{2\pi } w \bigl(d_{\mathbb {D}}\bigl(\tanh (\tau /2)e^{i\theta }, \tanh \bigl(\tau '/2 \bigr)e^{i\theta '} \bigr) \bigr) \\ &\quad {} \times S_{\mu }\bigl(v\bigl(\tau ',\theta ',t\bigr)\bigr)\sinh \bigl(\tau '\bigr)\,d\tau '\,d \theta '. \end{aligned} $$

Assuming as in Sect. 5.2.1 that $S(0)=0$, the basic state is $v=0$. The eigenvalue problem of the linearized operator is as follows:

32 $$ +\mu \int _{0}^{\omega } \int _{0}^{2\pi } w \bigl(d_{\mathbb {D}}\bigl(\tanh (\tau /2)e^{i\theta },\tanh \bigl(\tau '/2 \bigr)e^{i\theta '} \bigr) \bigr) \times v\bigl(\tau ',\theta '\bigr)\sinh \bigl(\tau '\bigr)\,d\tau '\,d\theta ', $$

where $\mu =S'(0)$. Since *Ω* is compact, we expect the spectrum to consist of isolated eigenvalues with finite multiplicity. Then, due to the invariance of *Ω* by rotations and reflections, the bifurcation problem enters into the classical framework of bifurcation with $O(2)$ symmetry.

In order to compute the bifurcation point and the corresponding eigenvectors, we need to determine a suitable basis of square integrable eigenfunctions of the Laplace–Beltrami operator in *Ω* with Dirichlet boundary conditions. By formula (26), we look for eigenfunctions of the form $e^{im\theta }\mathcal {P}^{m}_{l}(\cosh (\tau ))$ with $l=-1/2+i\rho $ (*ρ* real) and satisfying the boundary condition $\mathcal {P}^{m}_{l}(\cosh (\omega ))=0$. Here $\mathcal {P}^{m}_{l}$ are associated Legendre functions. It was shown in [21] that the boundary conditions are satisfied for a discrete set of values $l_{m,n}=-1/2+\rho _{m,n}$ of *l* such that $n\in \mathbb {N}^{*}$, $0< l_{m,n}< l_{m,n+1}$, and $\lim_{{n\rightarrow +\infty }} l_{m,n}=+\infty $. The corresponding eigenvalues of the Laplace–Beltrami operator are $\lambda _{m,n}=-l_{m,n}(l_{m,n}+1)$.

Moreover, the functions $\mathcal {P}^{m}_{l_{m,n}}$ are orthogonal for the measure $\sinh (\tau )\,d\tau $. We denote by $\mathcal {Y}^{m}_{n}(\tau )$ the normalized eigenfunctions.

In analogy with [8] for the spherical model, we may expend the connectivity function as

$$ \sum_{m=0}^{+\infty }\sum _{n\in \mathbb {N}^{*}}\widehat{w}_{m,n}\mathcal {Y}^{m} _{n}(\tau )\mathcal {Y}^{m}_{n}\bigl(\tau '\bigr)\cos \bigl(m\bigl(\theta -\theta '\bigr) \bigr). $$

Then the eigenvalue problem reduces to

$$ \lambda _{m,n}=-1+\mu \widehat{w}_{m,n} $$

with corresponding eigenvectors $\mathcal {Y}^{m}_{n}(\tau )\cos (m\theta )$ and $\mathcal {Y}^{m}_{n}(\tau )\sin (m\theta )$. The basic state becomes unstable at the critical value $\mu _{c}=\widehat{w}(m_{0},n_{0})^{-1}$, where $\widehat{w}(m_{0},n_{0})=\max_{m,n}\{\widehat{w}(m,n)\}$.

**Figure 14:**
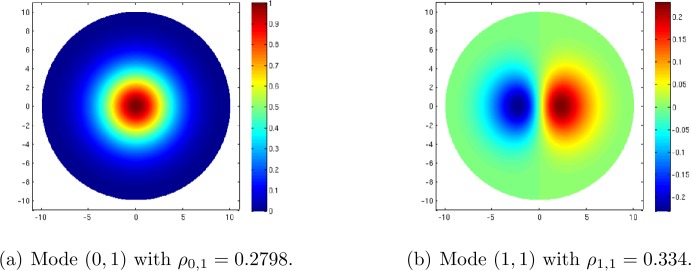
Plot of bifurcated solutions of (31) of the form $\mathcal {P}^{m}_{l_{m,n}}(\cosh (\tau ))\cos (m\theta )$ with $(m,n)=(0,1)$ and $(1,1)$

The relevant cases from the biological point of view are $(m_{0},n _{0})=(0,1)$ and $(1,1)$. In the first case the resulting bifurcated state behaves like $Y^{0}_{1}(\tau )$ (radial solution, hence no orientation preference), while in the second case it behaves like $Y^{1}_{1}(\tau )\cos (\theta -\varphi )$, where *φ* is an arbitrary phase (unimodal orientation). See Fig. 14 for a representation of these two states. The bifurcation analysis and the integration into the “spatialized” model, including the long range interactions, are provided in [21]. It was concluded there that for the spatially extended problem no new solution was obtained with regard to the “classical” model of [10]. This is not a surprise since the symmetries are the same. Nevertheless, the structure tensor model in *Ω* contains more information due to the additional, radial variable *τ*, which features selectivity of the signal.

## Conclusion

I conclude this survey by some remarks about the structure tensor model.

First, although some arguments can be found to support the biological relevance of this model, no experimental protocol has yet been designed to test the hypothesis. Next, one is still far from understanding in a satisfactory manner the bifurcation of solutions in the hyperbolic plane or Poincaré disc. In particular there is no evidence that the solutions which have been computed so far are physically or physiologically observable.

These difficulties were the reason why the model has not been further studied. Nevertheless, the methods which have been developed not only are interesting for their own, but could also be applied to other contexts of neurogeometry or physics. This was the main motivation for writing this review paper.

A final remark is that approximating the Poincaré disc with a disc *Ω* of finite size as in Sect. 5.2.3 simplifies considerably the bifurcation and stability analysis. The spatialized problem $\mathbb {R}^{2}\times \varXi $ was tackled in [21] in both cases: (1) with $\varXi =\varOmega $, (2) with $\varXi =\mathbb {D}$ and periodic octagonal tiling. Case 1 allows to extend the framework of spontaneous hallucinatory patterns as studied by [10], but it does not predict new hallucinatory patterns, on the contrary to case 2 in which new patterns have been found. However this work is quite technical and goes beyond the scope of the present survey.
